# Zinc excess impairs *Mycobacterium bovis* growth through triggering a Zur-IdeR-iron homeostasis signal pathway

**DOI:** 10.1128/spectrum.01069-23

**Published:** 2023-09-05

**Authors:** Xiaohui Li, Liu Chen, Yuankun Wang, Xiao Guo, Zheng-Guo He

**Affiliations:** 1 State Key Laboratory for Conservation and Utilization of Subtropical Agro-bioresources, Guangxi Research Center for Microbial and Enzyme Engineering Technology, College of Life Science and Technology, Guangxi University, Nanning, Guangxi, China; 2 College of Life Science and Technology, Huazhong Agricultural University, Wuhan, Hubei, China; Johns Hopkins University School of Medicine, Baltimore, Maryland, USA

**Keywords:** *Mycobacterium bovis*, zinc toxicity, iron homeostasis, transcription regulator, mycobacterial survival

## Abstract

Zinc excess is toxic to bacteria and, thus, represents an important innate defense mechanism of host cells, especially against mycobacterial infections. However, the signaling pathway triggered by zinc excess and its relationship with iron homeostasis remain poorly understood in mycobacteria. Here, we characterize a novel Zur-IdeR-iron homeostasis signaling pathway that modulates the growth of *Mycobacterium bovis* under zinc toxicity. We found that the regulator Zur interacts with the iron-homeostasis regulator IdeR, enhancing the DNA-binding ability of IdeR. Excess zinc disrupts this interaction and represses *ideR* transcription through Zur, which promotes the expression of iron uptake genes and leads to the accumulation of intracellular iron in *M. bovis*. The elevated iron levels lower the bacterial survival ability under excess zinc stress. Consistently, deleting *zur* hinders intracellular iron accumulation of *M. bovis* and enhances bacterial growth under stress, while silencing *ideR* impairs the growth of the wild-type and *zur*-deleted strains under the same conditions. Interestingly, both Zur and IdeR are conserved in bacteria facing zinc toxicity. Overall, our work uncovers a novel antimicrobial signal pathway whereby zinc excess disrupts iron homeostasis, which may deepen our understanding of the crosstalk mechanism between iron and zinc homeostasis in bacteria.

**IMPORTANCE:**

As a catalytic and structural cofactor of proteins, zinc is essential for almost all living organisms. However, zinc excess is toxic and represents a vital innate immunity strategy of macrophages to combat intracellular pathogens, especially against mycobacterial pathogens such as *Mycobacterium tuberculosis*, the causative agent of tuberculosis. Here, we first characterize an antibacterial signaling pathway of zinc excess and its relationship with iron homeostasis in *M. bovis*. We found that excess zinc inhibits the transcription of *ideR* and its DNA-binding activity through Zur, which, in turn, promotes the expression of iron uptake genes, causes intracellular iron accumulation, and finally impairs the bacterial growth. This study reveals the existence of the Zur-IdeR-iron homeostasis pathway triggered by zinc excess in *M. bovis*, which will shed light on the crosstalk mechanisms between zinc and iron homeostasis in bacteria and the antimicrobial mechanisms of host-mediated zinc toxicity.

## INTRODUCTION

Metal ions are essential for almost all living organisms because of their roles as catalytic and structural enzyme cofactors in numerous physiological processes (1, 2). However, they can be cytotoxic at high concentrations, leading to non-programmed cell death of eukaryotes, ferroptosis (3 – 5) and cuproptosis (6), and bacterial copper intoxication (7, 8). Zinc, a redox-inactive metal, is also toxic to bacteria in excess and, thus, represents an important innate defense mechanisms of host cells (9 – 14). During bacterial pathogen infections, free zinc is immediately accumulated inside macrophages and released into the infected phagosome. Subsequently, it is pumped into the bacterial cytoplasm to inhibit their growth, thereby preventing the spread of the infection (9, 11, 12, 14).

Recently, various mechanisms of bacterial resistance to zinc toxicity have been reported, including zinc uptake/efflux transporters and regulatory proteins (15 – 18). Under excess zinc stress, these regulators can sense environmental zinc levels to promote the expression of genes encoded zinc efflux transporters and inhibit zinc uptake gene expression. This ultimately lowers intracellular zinc contents, enhancing bacterial survival (19). Despite this, the antibacterial mechanisms of zinc toxicity have only been demonstrated in a few bacteria. For example, in *Streptococcus pneumoniae*, zinc competes with manganese for binding to pneumococcal surface adhesin A, leading to the inhibition of manganese acquisition (20). In *Bacillus subtilis*, zinc causes dysregulation of the PerR regulon, resulting in heme toxicity (21). In *Escherichia coli*, zinc increases intracellular iron levels, which disrupts the DNA-binding activity of CueR, the primary Cu detoxification transcription regulator, making the bacteria sensitive to copper (22).

Mycobacterial species, including *M. tuberculosis* (Mtb), are usually used to study bacterial adaptation mechanisms to host-mediated zinc toxicity (9, 15 – 18). Although the antimicrobial mechanisms of zinc excess remain unclear in mycobacteria, finely tuned crosstalk between iron and zinc homeostasis exists. For instance, Wagner et al. observed a significant increase in both iron and zinc levels within macrophages infected with pathogenic mycobacteria between 1 and 24 h post-infection (23). The ESX-3 system, one of five type VII secretion systems, is conserved and involved in both iron and zinc uptake in mycobacteria (24 – 28). Its expression is directly regulated by IdeR (29 – 31) and Zur (32). Interestingly, Zur is also required for heme-mediated iron uptake in Mtb (33). Moreover, zinc excess has been demonstrated to disturb iron homeostasis in *E. coli* (22). Therefore, we wonder whether zinc excess interferes with iron homeostasis in mycobacteria and how it occurs.

Considering the universality of zinc toxicity across mycobacterial species (9, 15 – 18), we used *M. bovis* BCG, a model strain for studying Mtb, to investigate the antimycobacterial mechanism of excess zinc. We found that zinc stimulates the expression of iron uptake genes by inhibiting *ideR* transcription and disrupting the interaction between IdeR and Zur in *M. bovis* BCG. This leads to increased levels of iron, ultimately impairing bacterial growth. Additionally, we observed that *zur* deletion reduces intracellular iron accumulation and enhances mycobacterial growth under excess zinc stress through IdeR. Therefore, our work characterizes a novel Zur-IdeR-iron signal pathway triggered by zinc toxicity in mycobacteria, which will provide a glimpse for the crosstalk mechanisms of metal ion homeostasis and the bacterial adaptation mechanisms to host-mediated zinc toxicity.

## RESULTS

### Excess zinc causes intracellular iron accumulation and growth inhibition of *M. bovis* BCG

**Fig 1:**
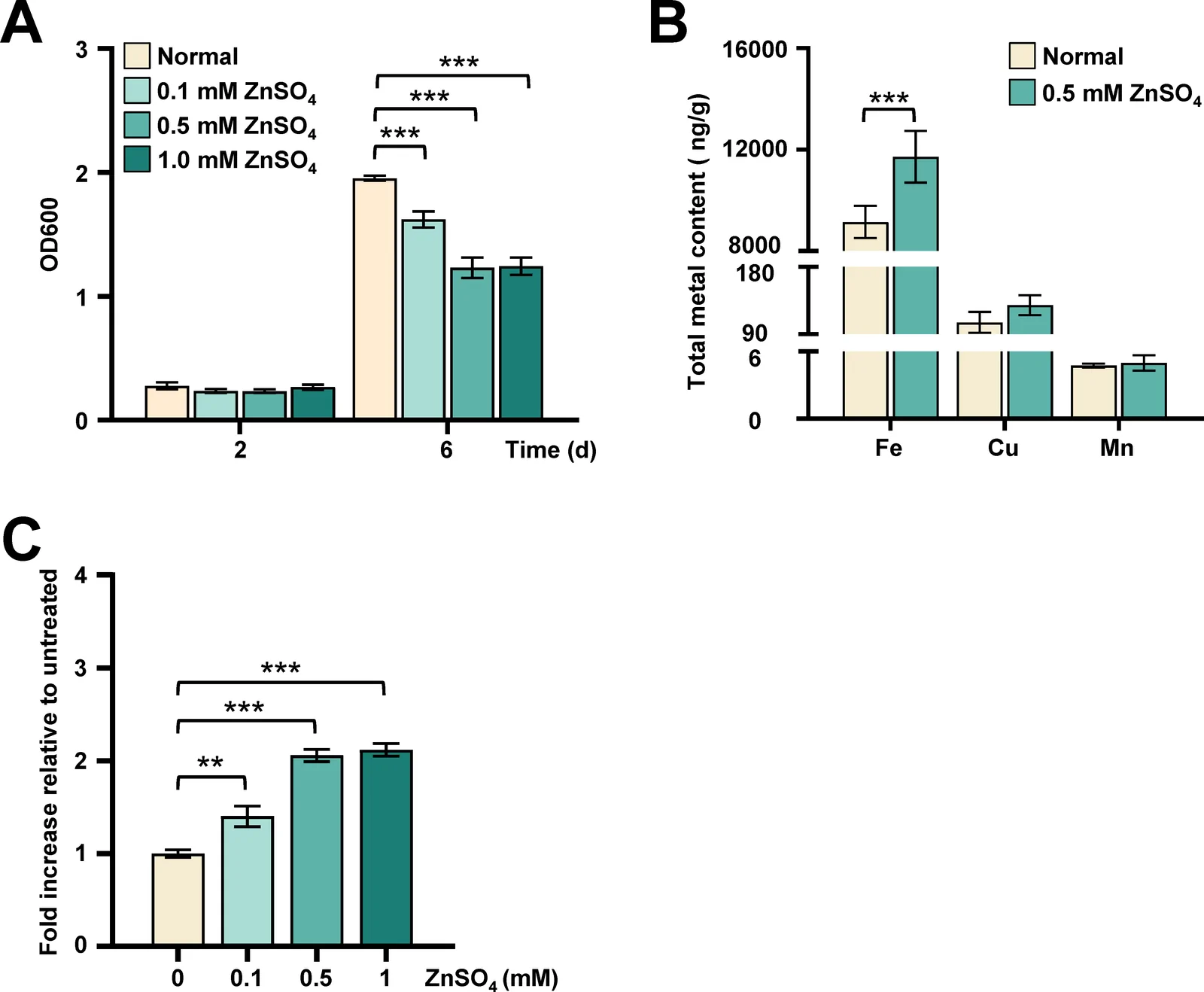
Zinc excess causes intracellular iron accumulation and inhibits the growth of *M. bovis* BCG. (**A**) Assays for the effects of different concentrations (0.1–1 mM) of zinc on the growth of the wild-type strain of *M. bovis* BCG. (**B**) The intracellular metal content of the wild-type strain was measured under 0.5 mM zinc by ICP-OES. Intra-bacterial metal content is expressed as nanograms of metal per gram of bacterial extract. (**C**) The effect of zinc on the intracellular iron level of the wild-type strain was analyzed by iron-detection reagent assays. The wild-type *M. bovis* BCG strain was cultured in 7H9 medium until reaching an OD600 of 0.8 and then treated with or without the indicated concentration of ZnSO_4_ for 24 h. Subsequently, the intracellular metal content in bacterial pellets was determined using ICP-OES or iron-detection reagent assays. *Error bars*, S.D. Statistical significance was calculated with Student’s *t*-test; **P < 0.01; ***P < 0.001.

Previous studies have shown that high concentrations of zinc can lead to the metalation of various non-zinc metalloenzymes (20 – 22, 34) and thereby impair bacterial growth. To test this, we used *M. bovis* BCG as a model and monitored their growth difference under different concentrations of zinc by turbidity. As shown in Fig. 1A, the wild-type (WT) strain exhibited inhibited growth at the increasing concentrations of zinc from 0.1 to 1 mM. Next, we investigated the effects of excess zinc on the content of other transition metals in *M. bovis* using inductively coupled plasma optical emission spectrometry (ICP-OES). As shown in Fig. 1B; Fig. S1A, intracellular levels of zinc and iron in the bacteria were separately increased ~29.5-fold and ~1.3-fold under 0.5 mM zinc stress, while the content of other metal ions, including copper and manganese, was not changed. To validate these observations, the *ctpG*-deleted *M. bovis* strain was used as a control, which is sensitive to excessive zinc (16). The intracellular metal content was then determined in both the wild-type strain and the *ctpG*-deleted strain when treated with and without 0.5 mM zinc. As shown in Fig. S1B, both strains exhibited significantly increased intracellular iron levels under stress, with the *ctpG*-deleted strain showing higher levels than that of the wild-type strain. In contrast, the copper contents in these strains showed no obvious difference under the same experiment conditions (Fig. S1B). Furthermore, we confirmed the results by measuring the total iron contents of the wild-type strains when treated with different concentrations of zinc using iron-detection reagent assays. We found that in the zinc-treated samples, intracellular iron content gradually increased with increasing zinc concentration up to approximately 2.2-fold compared to that of the untreated samples (Fig. 1C). Therefore, these results indicate that excess zinc causes intracellular iron accumulation and inhibits *M. bovis* BCG growth.

### Excess zinc activates the IdeR regulon in *M. bovis*

**Fig 2:**
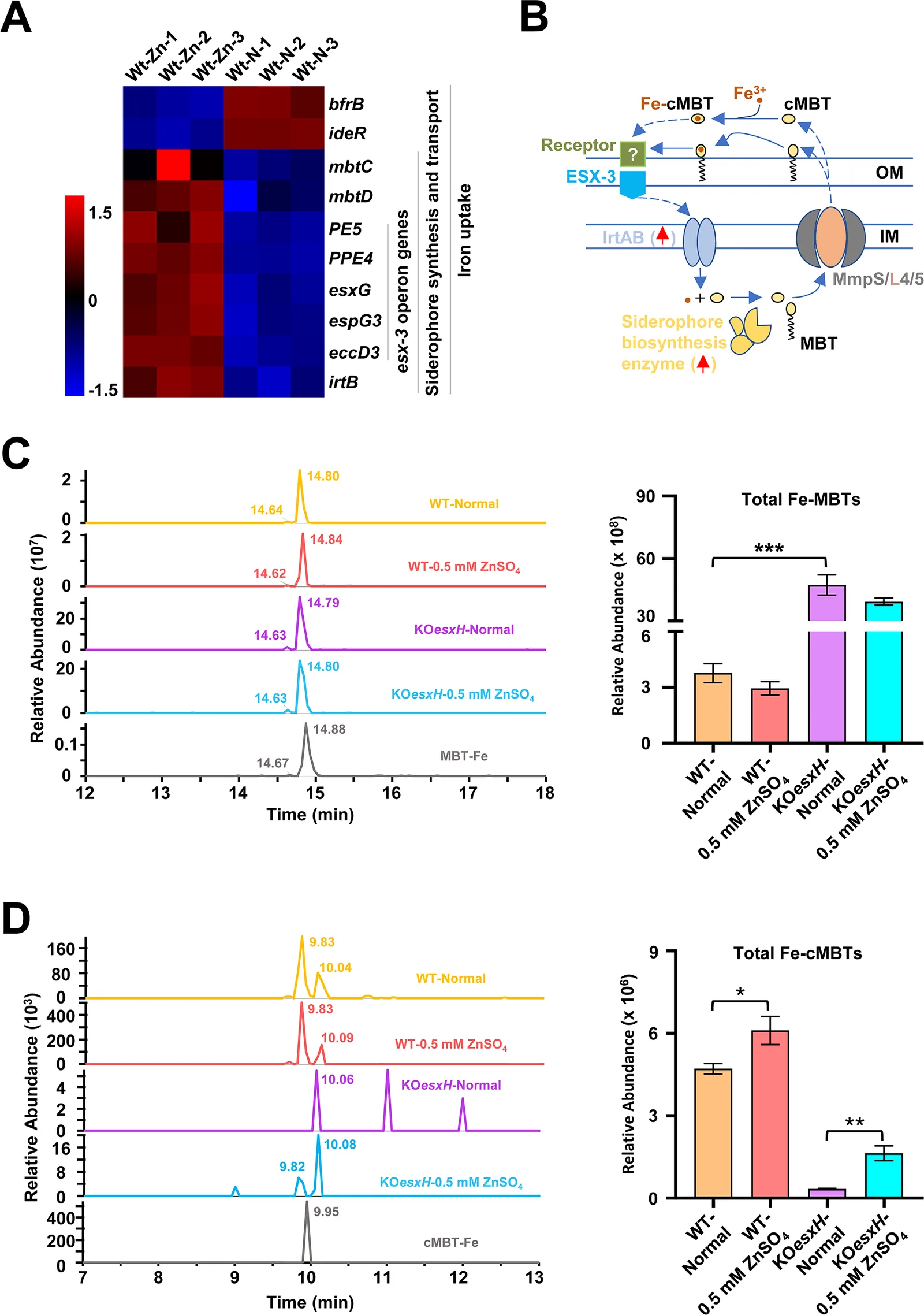
Zinc excess causes the increased levels of intracellular siderophore of *M. bovis* BCG. (**A**) Heat map showing the expression profiles of iron uptake genes in *M. bovis* BCG strains under 0.5 mM zinc stress. The gene expression data were obtained from reported RNA-Seq data (16), with a red-blue density display indicating the expression levels. Wt-N and Wt-Zn represent three biological replicates of differentially expressed genes (fold change > 1.5 and *P* < 0.05) in the wild-type strains treated without and with 0.5 mM zinc. (**B**) Schematic overview of the effects of zinc on the pathway of siderophore synthesis and transport (41), with the red arrows indicating the upregulated expression of genes mentioned in (**A**). (**C and D**) Quantitative analysis of the effects of excessive zinc on MBT (**C**) and cMBT (**D**) abundance in whole-cell lipid extracts of the wild-type strain. The *esxH*-deleted strain (KO*esxH*) was used as a control. *Error bars*, S.D. Statistical significance was calculated with Student’s *t*-test; *P < 0.05; **P < 0.01; ***P < 0.001.

To explore the mechanism that zinc excess affects intracellular iron level, we first focused on analyzing transcriptome data (16) from *M. bovis* BCG strains induced with 0.5 mM zinc for 24 h. We found that the expression of several genes involved in iron uptake, especially siderophore synthesis & import genes (*irtB*, *mbt* operon, and *esx-3* operon genes) (27, 35 – 38), was significantly upregulated in the bacteria under 0.5 mM zinc (Fig. 2A and B). In contrast, the expression of genes associated with iron store (*bfrB*) (31, 39) and *ideR* (31) was downregulated under the same experiment conditions (Fig. 2A). Furthermore, qRT-PCR results confirmed the transcriptome data. As shown in Fig. S2, the expression of the *esx-3* operon genes, *esxG/H*, and the positive control gene, *cmtR* (40), was progressively induced in *M. bovis* under 0.5 mM zinc from 4 to 24 h. These results indicate that excess zinc activates the IdeR regulon, causing increased expression of iron uptake genes and decreased expression of iron storage genes in *M. bovis*.

To further verify the observation, we determined the levels of siderophores (small molecules with extremely high affinity for iron (III), including mycobactins and carboxymycobactins) in wild-type *M. bovis* strains treated with and without 0.5 mM zinc by high-resolution mass spectrometry. The *esxH*-deleted *M. bovis* strain was used as a control, which has been reported to overproduce MBTs (mycobactins) in Mtb (27). Compared with the wild-type strain, the *esxH*-deleted strain showed no growth difference but demonstrated distinct orange pigmentation under normal conditions (Fig. S3) and accumulated ~12.5-fold MBTs within the cells (Fig. 2C; Fig. S4A and S4B), which is consistent with previous data (27). In zinc-treated samples, no obvious difference in MBTs content in both strains was observed compared to untreated samples (Fig. 2C; Fig. S4A and S4B). However, cMBTs (carboxymycobactins) levels in the wild-type and *esxH*-deleted strains were separately increased ~1.3-fold and 4.8-fold under zinc stress (Fig. 2D; Fig. S4C and S4D). These results indicate that excess zinc raises intracellular siderophore levels in *M. bovis*, consistent with both the increased expression of iron uptake genes (Fig. 2A) and the elevated levels of intracellular iron (Fig. 1B and C) under stress.

Collectively, these data suggest that zinc excess activates the IdeR regulon of *M. bovis* BCG.

### Activating the *ideR* regulon and increasing iron levels impair *M. bovis* growth under zinc toxicity

**Fig 3:**
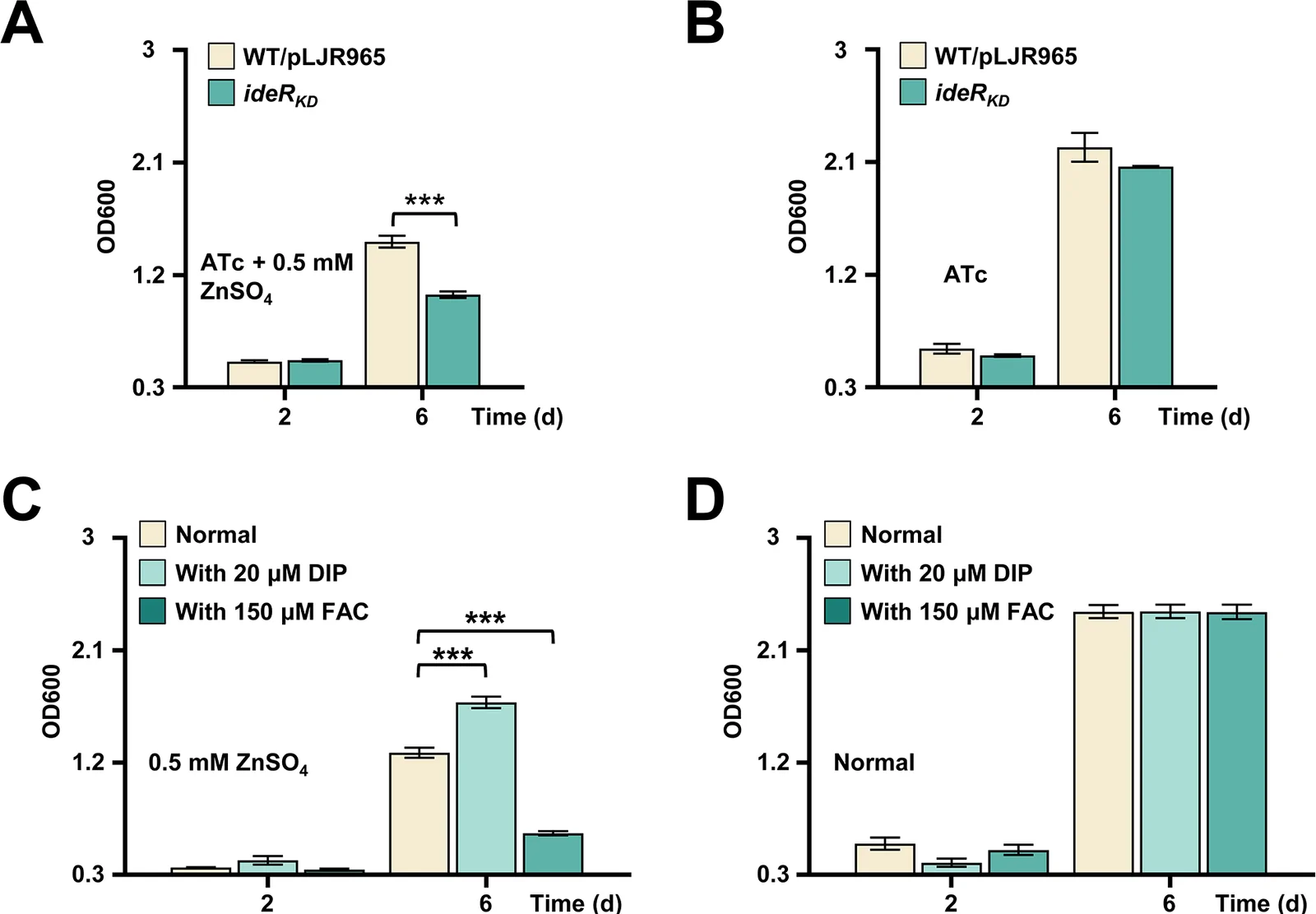
Disturbing iron homeostasis affects the growth of *M. bovis* BCG under excess zinc stress. (**A and B**) The growth difference between the wild-type and *ideR* silencing strains was determined in 7H9 medium supplemented with (**A**) and without (**B**) 0.5 mM zinc in the presence of 50 ng/mL ATc. sgRNA was designed to target the coding region of *ideR*, and a non-targeting sgRNA was used as a control. WT/pLJR965 represents the BCG/pLJR965 strain; *ideR_KD_* represents the BCG/pLJR965-*ideR* strain. (**C and D**) Assays for the effects of iron content on the growth of *M. bovis* BCG treated with (**C**) and without (**D**) 0.5 mM zinc. The wild-type strain was cultured twice in self-made low-iron medium to deplete intracellular iron before growth analysis. Subsequently, the iron-depleted cells were cultivated in low-iron medium at an initial OD600 of 0.1 supplemented with and without 20 µM 2,2′-dipyridyl (DIP) and 150 µM ammonium ferric citrate (FAC), respectively. *Error bars*, S.D. Statistical significance was calculated with Student’s *t*-test; ***P < 0.001.

Next, we silenced the expression of *ideR* in *M. bovis* BCG by CRISPRi-mediated gene knockdown strategy (42). As shown in Fig. S5, *ideR* expression was downregulated ~4.4-fold in the *ideR*_KD_ strain in the presence of 50 ng/mL anhydrotetracycline hydrochloride (ATc) compared with that of the wildtype strain. Meanwhile, the expression of iron uptake genes (*esxG/H*, *irtA*, *mbtD*, and *mmpL4*) was significantly upregulated. By contrast, the expression of the control gene *zur* was not affected under the same experiment conditions. Subsequently, the growth difference between the wild-type and *ideR* silencing strains was determined in the presence and absence of 50 ng/mL ATc and 0.5 mM zinc stress. As shown in Fig. 3A, the *ideR*_KD_ strain grew much slower than that of the wild-type strain in the presence of ATc and zinc stress at 6 days. Under ATc induction, the *ideR*_KD_ strain showed only slightly impaired growth compared to the wildtype strain (Fig. 3B). However, no growth difference between these strains was observed under normal conditions (Fig. S6). These results indicate that activating the *ideR* regulon by silencing *ideR* expression reduces the survival ability of *M. bovis* BCG under stress.

To further test this result, the wild-type *M. bovis* BCG strain was iron-depleted by culturing them in self-made low-iron 7H9 medium. The iron-depleted cells were then cultured at an initial OD600 of 0.1 in the low-iron medium supplemented with 150 µM ferric ammonium citrate (150 µM FAC, the normal iron concentration in 7H9 medium) and 20 µM 2,2′-dipyridyl (DIP, an iron chelator), treated with and without 0.5 mM zinc, and the growth difference was determined. As shown in Fig. 3C, under stress, the addition of DIP into the medium significantly promoted bacterial growth at 6 days, while FAC addition obviously impaired mycobacterial growth. By contrast, under normal conditions, DIP but not FAC addition slightly inhibited *M. bovis* growth at 2 days (Fig. 3D). These results suggest that activating the *ideR* regulon and increasing intracellular iron levels impair *M. bovis* growth under zinc toxicity.

### Excess zinc neutralizes the Zur-mediated promotion of the DNA-binding activity of IdeR

**Fig 4:**
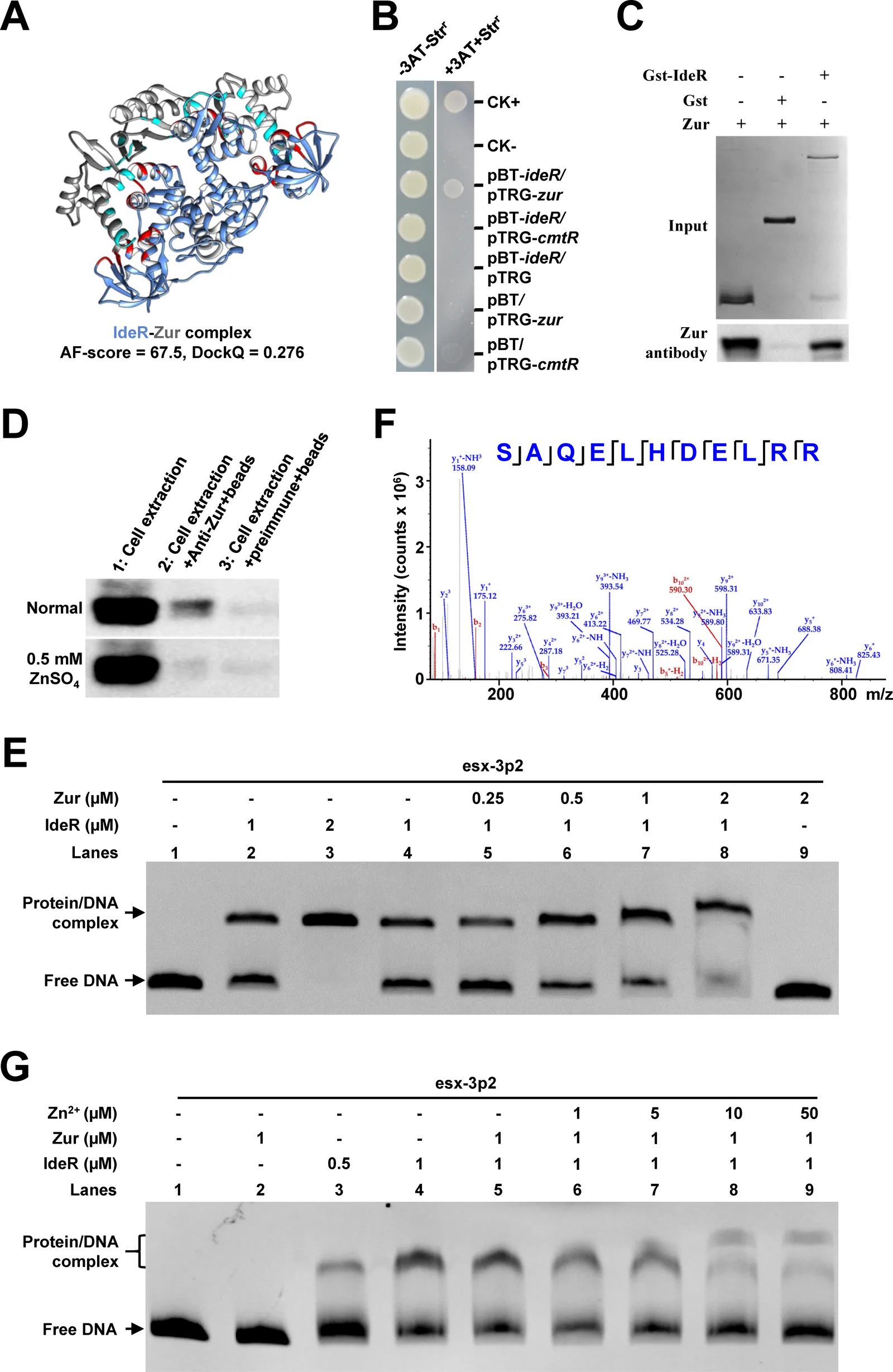
Zinc disrupts the interaction between IdeR and Zur. (**A**) The IdeR-Zur complex was predicted by AlphaFold v2.2.0. The structure of IdeR and Zur was separately marked in cornflower blue and dim gray. DockQ ≥ 0.23 indicates a certain level of accuracy in the prediction of the interface of the heterologous protein complex modeled by AlphaFold-Multimer. The potential interaction regions analyzed by UCSF Chimera software were marked in cyan and red. (**B**) Bacterial two-hybrid assays for the interaction between Zur and IdeR. Reporter strains of *E. coli* containing different recombinant plasmids were spotted on the plate in the presence or absence of streptomycin (Str) and 3-amino-1,2,4-triazole (3AT). The regulator gene, *cmtR*, was used as a control. The positive control, CK+, contains the co-transformant plasmids, pBT-LGF2 and pTRG-Gal11p, while the negative control, CK−, contains the empty vectors pBT and pTRG. (**C**) GST pull-down assays for the interaction between Zur and IdeR. The GST tag protein was used as a control, and the interaction was further confirmed by western blot with Zur antibody. (**D**) Co-IP assays for the interaction between Zur and IdeR in *M. bovis* BCG. A recombinant strain containing an *ideR-flag* expression plasmid was cultivated in 7H9 medium until reaching an OD600 of 1.0 and then treated without (top panel) and with (bottom panel) 0.5 mM zinc. After treatment, the cells were harvested, resuspended, and lysed. Protein A beads were conjugated with an antibody raised against Zur. The samples were analyzed by Western blot using anti-Flag antibody. (**E**) Electrophoretic mobility shift assay (EMSA) for studying the effects of Zur on the DNA-binding activity of IdeR. The esx-3p2 DNA substrate was co-incubated with the indicated quantities of IdeR (lanes 2–4), Zur (lane 9), and both (lanes 5–8). (**F**) NanoLC-MS identified the peptide of Zur in the shifted band corresponding to the IdeR-Zur-DNA complex, whose amino acid sequence was SAQELHDELRR. (**G**) EMSA for studying the effect of zinc on the DNA-binding activity of IdeR in the presence of Zur.

The results so far indicate that excess zinc leads to iron accumulation and growth inhibition of *M. bovis* by activating the IdeR regulon. However, the mechanism by which zinc activates this regulon remains unclear. Previous works have shown that Zur is a zinc homeostasis regulator in mycobacteria (32, 43) and is essential for heme-mediated iron absorption in Mtb (33). Collectively, these results imply the existence of a functional connection between Zur and IdeR. To test this hypothesis, we first used AlphaFold 2.0 (44) to predict their interaction. As shown in Fig. 4A, the Zur-IdeR complex demonstrated a high confidence score [model confidence score (AF-score) = 0.675] and a credible interaction interface (DockQ = 0.276), indicating that Zur may potentially interact with IdeR. To verify this interaction, bacterial two-hybrid assays were performed. As shown in Fig. 4B, the co-transformant containing *zur/ideR* was cultured successfully in the screening medium, while that containing *cmtR/ideR* did not grow. Meanwhile, the controls containing *zur/ideR* alone did not grow under the same conditions. These results indicate that IdeR interacts specifically with Zur. Furthermore, pull-down experiments confirmed the physical interaction, with Zur binding to GST-IdeR but not GST tag (Fig. 4C). To assess the physiological significance of the interaction, a co-immunoprecipitation (Co-IP) experiment was conducted in *M. bovis* BCG by Protein A beads conjugated with an antibody raised against Zur. An obvious and specific hybridization signal was detected (lane 2) and the signal band size matched the size of cell extraction of IdeR-Flag (lane 1) (Fig. 4D, top panel). However, no signal was detected when Protein A beads were not conjugated with the anti-Zur antibody (lane 3). Collectively, these results suggest that Zur physically interacts with IdeR in *M. bovis* BCG, both *in vitro* and *in vivo*.

To investigate the effect of Zur on IdeR activity, we first investigated the DNA-binding ability of IdeR and Zur by electrophoretic mobility shift assays (EMSAs). As shown in Fig. S7A and SB, both Zur and IdeR exhibited strong binding affinity with the esx-3 operon promoters, esx-3p1 and esx-3p2, respectively. Next, the DNA-binding ability of IdeR was examined in the presence of Zur. As shown in Fig. 4E, IdeR bound well with the promoter esx-3p2 (lanes 2–4), while Zur did not (lane 9); with the addition of increasing levels (0.25–2 μM) of Zur into the IdeR reaction mixture, the levels of shifted bands corresponding to the specific IdeR and esx-3p2 DNA complex were gradually increased (lanes 5–8). Subsequently, the protein component in the shifted band corresponding to the potential Zur-IdeR-DNA complex was analyzed by mass spectrometry. As shown in Fig. 4F; Fig. S8A, peptide fragments of both Zur and IdeR were identified in the shift, indicating that Zur interacts with IdeR, enhancing the DNA-binding activity. However, no obvious effects were observed in the levels of shifted bands corresponding to the complex when the same levels (0.25–2 μM) of CmtR were added into the IdeR reaction mixture (Fig. S8B).

To investigate the effect of zinc on the DNA-binding abilities of Zur and IdeR, EMSAs were performed. As shown in Fig. S9A, the levels of the shifted band of the Zur and esx-3p1 complex were gradually increased with the addition of 5–50 μM zinc into the reaction mixture (lanes 3–5). In contrast, no obvious effect was observed in those of the IdeR and esx-3p2 complex when the same concentrations of zinc were added into the IdeR reaction mixture (Fig. S9B). To study the effect of zinc on the Zur and IdeR interaction, a Co-IP experiment was conducted in *M. bovis* BCG strain treated with and without 0.5 mM zinc. As shown in Fig. 4D, the level of the Zur-IdeR complex relative to the IdeR level in the cell was significantly decreased under zinc treatment compared to that under normal conditions. Furthermore, we evaluated the effect of zinc on the DNA-binding activity of IdeR in the presence of Zur by EMSA. As shown in Fig. 4G, IdeR, instead of Zur (lane 2), bound well with esx-3p2 (lanes 3–4). Notably, upon the addition of 1–50 μM zinc into the mixtures, the shifted band levels of the complex gradually decreased in the presence of Zur (lanes 5–9). These results indicate that high concentrations of zinc attenuate the Zur-mediated promotion of IdeR’ DNA-binding activity.

Collectively, these results indicate that Zur physically interacts with IdeR and enhances the DNA-binding ability of IdeR, but excess zinc lowers the Zur-mediated promotion of the DNA-binding activity of IdeR.

### Deletion of *zur* inhibits intracellular iron accumulation and enhances *M. bovis* survival under zinc toxicity

**Fig 5:**
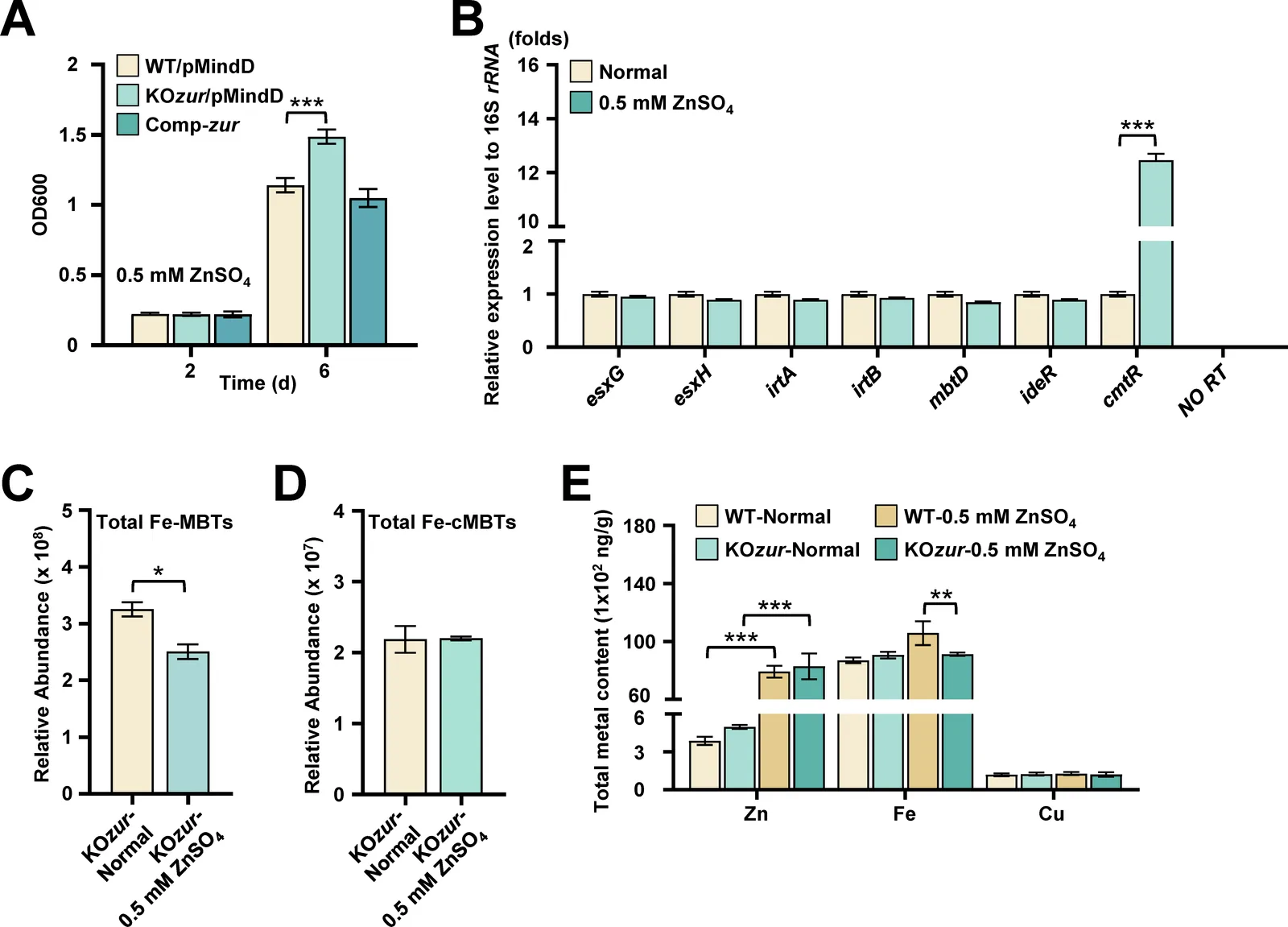
Zur regulates intracellular iron accumulation and the growth of *M. bovis* BCG under excess zinc stress. (**A**) Assays for studying the effect of *zur* deletion on the growth of *M. bovis* BCG under 0.5 mM zinc stress. WT/pMindD represents the BCG/pMindD strain; KO*zur*/pMindD represents the BCG *zur::hyg*/pMindD strain; Comp-*zur* represents the BCG *zur::hyg*/pMindD-*zur* strain. (**B**) qRT-PCR analysis of the expression of the IdeR regulon in the *zur*-deleted strain (KO*zur*) of *M. bovis* BCG upon exposure to 0.5 mM zinc for 24 h. The untreated strain and the reported zinc-induced gene (*cmtR*) (40) were separately used as controls. (**C and D**) Quantitative analysis of MBT (**C**) and cMBT (**D**) abundance in the whole-cell extracts of KO*zur* under 0.5 mM zinc stress. (**E**) Intracellular iron contents in the WT strain and the *zur*-deleted strain (KO*zur*) were determined by ICP-OES under 0.5 mM zinc stress. The data on intracellular metal contents of the wild-type strain were from Fig. S1B. *Error bars*, S.D. Statistical significance was calculated with Student’s *t*-test; *P < 0.05; **P < 0.01; ***P < 0.001.

To explore the role of Zur in *M. bovis* resistance to zinc toxicity, we constructed *zur* deletion and complementation strains and then determined their growth difference under 0.5 mM zinc stress. As shown in Fig. 5A, the growth of the *zur*-deleted strain was much better than that of the wild-type strain at 6 days of stress, while no obvious growth difference was observed between the wild-type strain and the *zur* complementation strain under the same experiment conditions. By contrast, under no zinc stress, the *zur*-deleted strain exhibited slight growth inhibition compared with the wild-type strain, and the *zur* complementation strain showed no obvious growth difference (Fig. S10). These results indicate that *zur* deletion enhances mycobacterial resistance to zinc toxicity in *M. bovis*.

Since Zur interacts with IdeR, which regulates iron homeostasis and mycobacterial resistance to zinc toxicity, we investigated the regulatory effect of Zur on the expression of iron uptake genes using qRT-PCR assays. As shown in Fig. S11, *esxG/H* expression was significantly upregulated in the *zur*-deleted strain, while the expression of *irtA/B*, *mbtD*, and *ideR* was not obviously changed. Furthermore, we examined the expression of these genes in the *zur-*deleted strains under 0.5 mM zinc stress. As shown in Fig. 5B, the expression of iron uptake genes was not affected in the *zur-*deleted strain under stress. By contrast, the expression of a control gene, *cmtR*, was significantly induced. Consistently, MBT levels in the *zur*-deleted strain were slightly decreased under 0.5 mM zinc stress compared to those under normal conditions (Fig. 5C; Fig. S12A and S12B); However, no obvious difference in cMBTs contents was observed under the similar experiment conditions (Fig. 5D; Fig. S12C and S12D). These results, combined with the above-mentioned data (Fig. 2; Fig. S2), indicate that the induction effects of zinc on the expression of iron uptake genes and siderophore level are dependent on Zur.

Next, the intracellular iron contents of the wild-type and *zur*-deleted strains were determined in the presence and absence of 0.5 mM zinc. As shown in Fig. 5E, intracellular zinc levels in both strains were significantly increased under stress. Meanwhile, the wild-type strain exhibited a higher level of intracellular iron than that of the *zur*-deleted strain under stress. However, the intracellular copper content between these strains displayed no difference under the same experiment conditions.

Taken together, these results indicate that *zur* deletion lowers the inductive effects of zinc on the expression of iron uptake genes, inhibits intracellular iron accumulation, and enhances *M. bovis* survival under zinc toxicity.

### The Zur-mediated resistance of *M. bovis* to zinc toxicity depends on IdeR

**Fig 6:**
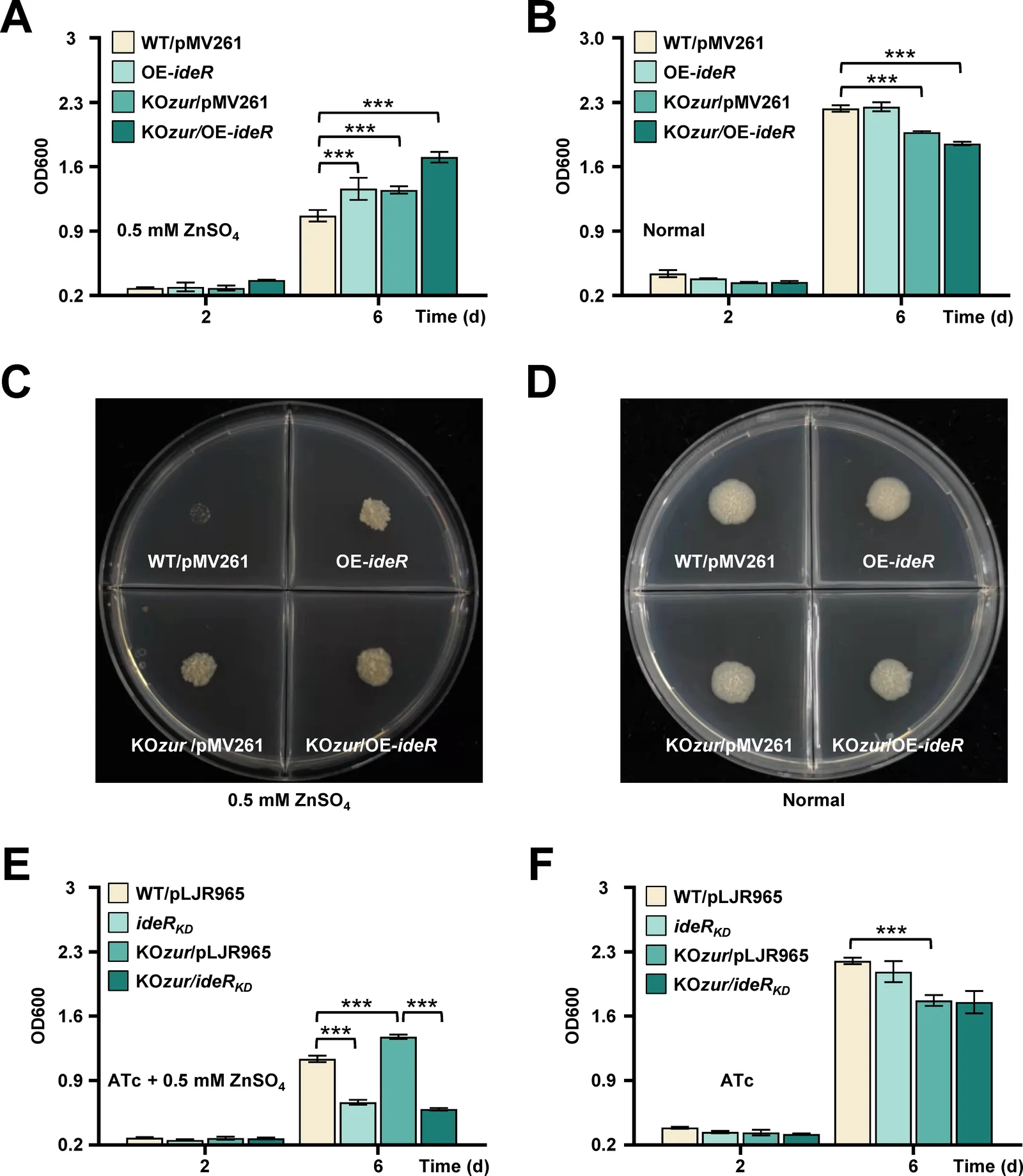
The Zur-mediated regulation of *M. bovis* resistance to zinc toxicity depends on IdeR. (**A and B**) Assays for studying the effects of *ideR* overexpression on the growth of the wild-type and *zur*-deleted *M. bovis* BCG strains in the presence (**A**) and absence (**B**) of 0.5 mM zinc. (**C and D**) Analysis of the sensitivity of recombinant *M. bovis* BCG strains to excess zinc. Recombinant strains were cultured in 7H9 medium up to an OD600 of 0.8 and spotted on 7H10 solid medium supplemented with (**C**) and without (**D**) 0.5 mM zinc, followed by culture at 37°C for 12 days and photographic analysis. (**E and F**) Assays for studying the effects of *ideR* silencing on the growth of the wild-type and *zur*-deleted strains treated with (**E**) and without (**F**) 0.5 mM zinc stress in the presence of 50 ng/mL ATc. WT/pMV261 represents the BCG/pMV261 strain; OE-*ideR* represents the BCG/pMV261-*ideR* strain; KO*zur*/pMV261 represents the BCG *zur::hyg*/pMV261 strain; KO*zur*/OE-*ideR* represents the BCG *zur::hyg*/OE-*ideR* strain; KO*zur*/pLJR965 represents the BCG *zur::hyg*/pLJR965 strain; KO*zur*/*ideR*_KD_ represents the BCG *zur::hyg*/pLJR965-*ideR* strain. *Error bars*, S.D. Statistical significance was calculated with Student’s *t-*test; ***P < 0.001.

To elucidate the dependence between IdeR and Zur in regulating mycobacterial resistance to zinc toxicity, we constructed an *ideR*-overexpression strain and overexpressed *ideR* in the *zur*-deleted strain and then determined their growth difference under 0.5 mM zinc stress. As shown in Fig. 6A, the *zur*-deleted strain and the *ideR* overexpression strain grew much better than the wild-type strain after 6 days of stress. When *ideR* was overexpressed in the *zur*-deleted strain, the recombinant strain exhibited better growth under stress compared with the wild-type strain. By contrast, deletion of *zur* but not *ideR* overexpression slightly impaired mycobacterial growth under no zinc stress (Fig. 6B; Fig. S8 and S13). To verify the observation, we tested their growth difference on 7H10 solid medium supplemented with and without 0.5 mM zinc. As shown in Fig. 6C, the *ideR* overexpression strain and the *zur*-deleted strain showed better growth compared to the wild-type strain under stress, and *ideR* overexpression obviously promoted the growth of the *zur*-deleted strain at the same experiment conditions. By contrast, no obvious growth difference was observed among these strains under no treatment condition (Fig. 6D). These results indicate that IdeR enhances the survival ability of the wild-type strain and the *zur*-deleted strain under zinc stress.

Next, we determined the effects of *ideR* silencing on the growth of the wildtype and *zur*-deleted strains treated with and without 50 ng/mL ATc and 0.5 mM zinc. As shown in Fig. 6E, silencing the expression of *ideR* significantly impaired the growth of the wild-type and *zur*-deleted strains in the presence of ATc and zinc at 6 days. However, *ideR* silencing only slightly inhibited mycobacterial growth under ATc induction (Fig. 6F), whereas the *zur*-deleted strain displayed modestly impaired growth regardless of the presence of ATc (Fig. 6F; Fig. S13). These results demonstrate that the effect of Zur on *M. bovis* resistance to zinc toxicity is dependent on IdeR.

### Zur and IdeR are conserved in bacteria confronting with zinc toxicity

Both IdeR and Zur regulate mycobacterial resistance to zinc toxicity. We wonder if these regulators are conserved in bacteria that have been reported to confront with zinc toxicity. Therefore, the phylogeny analysis of their homologs was conducted. As shown in Fig. S14, both Zur and IdeR are conserved and appear to be phylogenetically coevolved among these strains. Interestingly, *E. coli* (DE3) growth was inhibited when *zur* and *ideR* were co-expressed (Fig. S15, right panel). Subsequently, we investigated the co-expression of *zur* and *ideR* effects on *M. bovis* BCG growth. As shown in Fig. S15, mycobacterial growth (left panel) was inhibited as the co-expression occurred. Collectively, these results indicate that the biological roles of Zur and IdeR may be conserved in these bacteria.

## DISCUSSION

**Fig 7:**
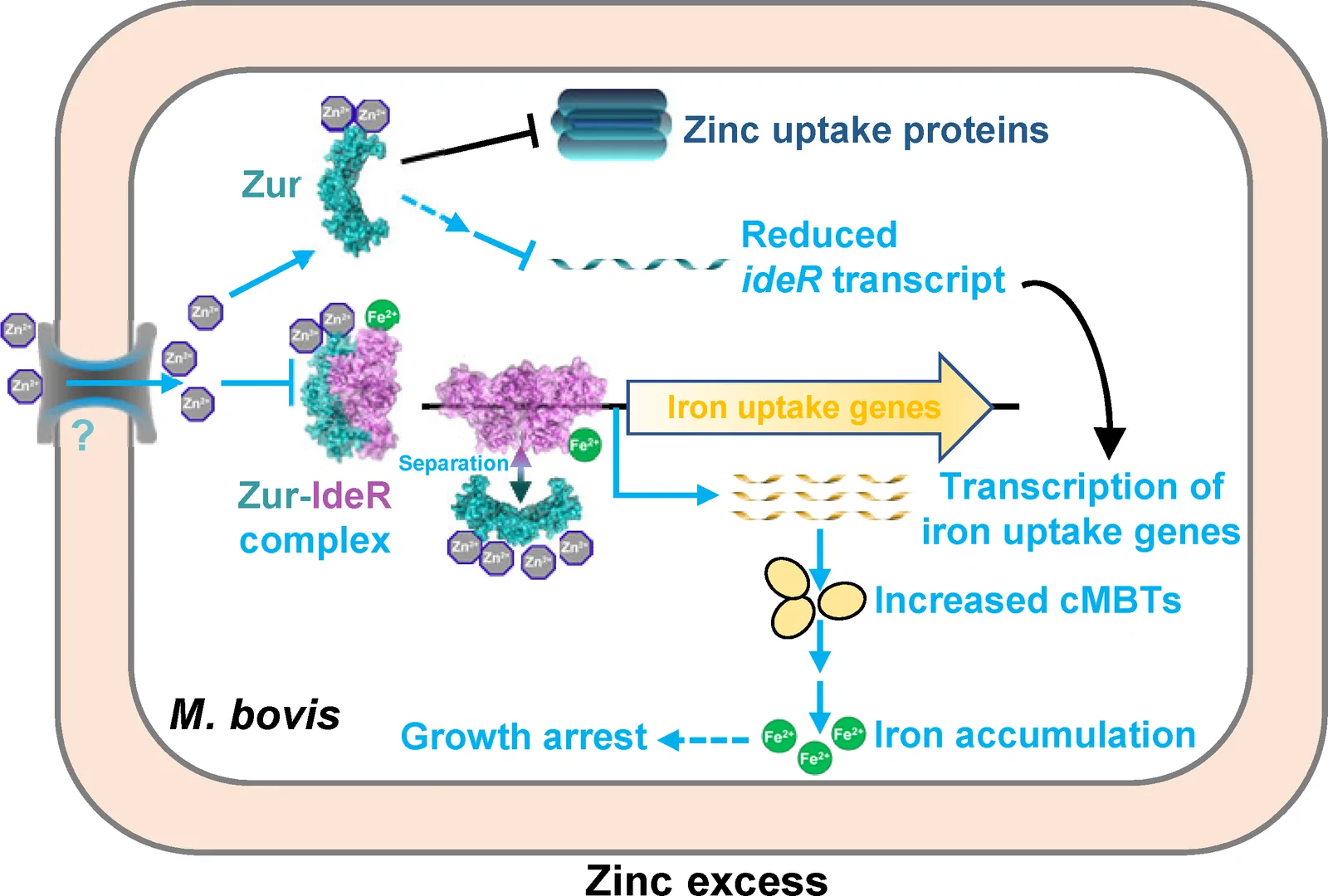
Cartoon summarizing the antimicrobial signal pathway of zinc excess in *M. bovis*. Upon exposure to zinc excess, zinc stimulates the expression of iron uptake genes (*mbt* operon, *irtA/B*, and *esx-3* operon) by inhibiting the negative regulation of the Zur-IdeR complex and *ideR* transcription through Zur, which causes intracellular iron accumulation of *M. bovis*. Subsequently, the increased iron impairs bacterial growth. The black lines represent the previously reported data, and the blue lines represent those in this study.

Zinc excess is used as a host innate immunity strategy to combat the infected intracellular pathogens, especially against mycobacterial pathogens like Mtb and *M. bovis*. However, the antibacterial mechanisms of excess zinc in these mycobacteria remain poorly understood. In this study, we used *M. bovis* BCG as a model and identified a novel signal pathway and antimicrobial mechanism of zinc excess in *M. bovis*. Our data are consistent with the model (Fig. 7) in which excess zinc inhibits *ideR* transcription through Zur and disrupts the Zur and IdeR interaction, de-repressing the inhibitory role of IdeR on the expression of iron uptake genes (*mbt/esx-3/irtAB* operon), causing intracellular iron accumulation and finally impaired bacterial growth. Interestingly, in the pathway, both Zur and IdeR are conserved in bacteria that face zinc toxicity. Therefore, our findings revealed the existence of a novel Zur-IdeR-iron homeostasis signaling pathway and a possible conserved antimicrobial mechanism triggered by excess zinc in mycobacteria. This work will provide new insights into the metal-mediated defense mechanisms of host cells.

The crosstalk between zinc and iron homeostasis has been reported in several bacteria, but the specific regulatory mechanisms are poorly understood. Previous studies have shown that excess zinc leads to the mis-metalation of non-Zn metalloproteins owing to its high affinity with these proteins. For example, in *B. subtilis*, zinc may disrupt PerR binding to iron or manganese, causing dysregulation of the PerR regulon and heme toxicity (21). In *E. coli*, zinc excess stimulates intracellular iron accumulation, leading to bacterial sensitivity to copper (22). Remarkably, mycobacteria have a finely tuned coordination between iron and zinc, with Zur and ESX-3 involved in both zinc and iron absorption (25, 32, 33). Intracellular zinc and iron levels in Mtb were increased within the infected macrophage phagosomes (23). In the present study, we found that excess zinc stimulates intracellular iron accumulation in *M. bovis*. Mechanically, excess zinc de-represses the inhibition effects of the IdeR-Zur complex and inhibits *ideR* transcription through Zur, which promotes the expression of iron uptake genes and finally causes intracellular iron accumulation. Therefore, our study first reveals a regulatory mechanism of crosstalk between zinc and iron homeostasis in bacteria.

In this study, we showed that *zur* deletion enhances mycobacterial resistance to zinc toxicity in *M. bovis* BCG. Previous work has shown that Zur regulates the expression of zinc uptake genes by sensing environmental zinc, which helps maintain zinc homeostasis in mycobacteria (32). In *Mycobacterium smegmatis*, the deletion of *zur* inhibits mycobacterial growth and resistance to zinc toxicity (17). Here, we also observed that Zur is able to sense environmental zinc and regulate the expression of zinc uptake genes *esxG/H* in *M. bovis* BCG and that the lack of *zur* impairs mycobacterial growth. However, *zur* deletion promotes *M. bovis* resistance to zinc toxicity by affecting iron homeostasis. There are several possible reasons for this functional difference in Zur. First, *M. bovis* is an intracellular pathogen that faces a more constant zinc environment compared with the environmental microorganism *M. smegmatis*. Second, the zinc-tolerance ability between these strains is different, with *M. bovis* more sensitive than *M. smegmatis* under zinc stress (16, 17). Third, the regulation mechanisms of zinc homeostasis differ between these strains. In *M. smegmatis*, the *smtB-zur* operon essential for zinc metabolism is directly regulated by both Zur and SmtB (17), while the operon seems to be solely affected by SmtB in Mtb (45). Meanwhile, the experiment methods used are different. In the previous research, zone of inhibition (ZoI) assays was conducted to determine zinc stress susceptibility using thawed cells and lysogeny broth (LB) medium in the presence of 25–100 mM zinc (17), which completely differs from the approach in this study. Moreover, Zur may play different roles in the resistance of mycobacterial species to zinc toxicity by regulating intracellular zinc or iron homeostasis.

An interesting observation was that the bands of the Zur-IdeR-DNA complex shift upward with increasing concentrations of zinc or Zur. In this study, we confirmed that Zur and IdeR interact with each other *in vitro* and *in vivo* in *M. bovis* BCG. Additionally, we identified the existence of the Zur-IdeR-DNA complex through mass spectrometry. There may be two reasons why the mobility of this complex changes in the presence of Zur. First, Zur and IdeR may form different oligomers that bind to DNA, leading to an increase in the shifted band formed. Second, after Zur and IdeR form the complex, they may bind to different sites on the DNA, causing changes in the size and shape of the complex, which, in turn, leads to the bands keep shifting up. Zinc may affect the mobility of the Zur-IdeR-DNA complex by influencing the oligomerization of the Zur-IdeR complex, its DNA binding activity, and even the charge distribution of the complex. As a support, Zur contains multiple high-affinity zinc-binding sites, and its conformation and affinity for DNA differ depending on the concentration of Zn (46). Interestingly, Zur contains two CxxC motifs that are crucial for both Zn binding and Zur oligomerization (43). Therefore, Zur may be mainly responsible for changing the mobility of the complex through zinc. However, further experiments are necessary to verify the exact reasons behind these changes.

Although a growing number of studies have been reported on bacterial resistance to zinc toxicity, the antibacterial mechanisms of this stress have only been identified in a few bacteria. For example, in *S. pneumoniae*, excess zinc causes mis-metalation of PsaA and inhibits manganese uptake, leading to bacterial sensitivity to oxidative stress (20). In *E. coli*, excess zinc stimulates intracellular iron accumulation and reduces bacterial tolerance to copper toxicity (22). In *B. subtilis*, excessive zinc causes dysregulation of the PerR regulon, causing heme toxicity (21). Mycobacterial species are usually used to study the mechanisms of adaptation to zinc toxicity, but the specific antibacterial mechanism of zinc excess remains largely obscure. In this study, we found that zinc excess stimulates iron accumulation of *M. bovis* and, thus, inhibits the bacterial growth. Two possible explanations exist for the concrete antibacterial mechanism. First, excess iron can oxidize bacterial biological components such as lipids, proteins, and nucleic acids due to its redox nature (34). As a support, the expression of multiple redox genes, such as the *nuoG* operon (47) and *whiB* genes (48), was induced in *M. bovis* BCG under zinc excess (16). Second, siderophore accumulation triggered by excess zinc may be toxic to the bacteria. A previous study reported that the deletion of *mmpS4/S5* encoded siderophore exporters in Mtb resulted in the accumulation of cell-associated siderophores, which impaired bacterial growth (41). Similarly, we observed that zinc excess significantly inhibited *mmpL/S5* expression (16) and caused cell-associated cMBT accumulation in *M. bovis*. Therefore, the antibacterial mechanisms of excess zinc may be related to siderophore toxicity and iron-mediated ROS damage, but further investigations are required.

In summary, we characterized a nove Zur-IdeR-iron signaling circuit triggered by excess zinc in *M. bovis*. We found that Zur physically interacts with IdeR. Under zinc excess stress, zinc promotes the expression of iron uptake genes by inhibiting the negative regulation of the Zur-IdeR complex and *ideR* transcription through Zur, leading to intracellular iron accumulation and growth inhibition of *M. bovis*. Our results combined with previous study (22) reveal a conserved antibacterial mechanism of zinc toxicity through disturbing iron homeostasis and will deepen our understanding of metal crosstalk mechanisms in bacteria and the metal-mediated defense strategies of host cells.

## MATERIALS AND METHODS

### Bacterial strains and culture conditions

The bacterial strains used in this study are listed in Table S1. *E. coli* strains were cultivated in LB medium at 37°C. *M. bovis* BCG strains were grown at 37°C in 7H9 medium supplemented with 0.2% glycerol, 0.05% Tween 80%, and 10% OADC (oleic acid, albumin, dextrose, and catalase) or on Middlebrook 7H10 agar supplemented with 0.5% glycerol and 10% OADC. All strains cultured in the liquid medium mentioned above were under shaking conditions at 160 rpm at 37°C. If necessary, antibiotics were added at the following concentrations: kanamycin, 25 µg/mL; hygromycin, 100 µg/mL.

For studying the effect of iron on mycobacterial resistance to zinc toxicity, self-made low-iron 7H9 medium was used as described previously (33). Briefly, *M. bovis* BCG was cultured in 7H9 medium until an OD600 of 1.5 was reached. The cells were then iron-depleted twice with self-made low-iron 7H9 medium supplemented with 0.5% glycerol, 0.2% casamino acids, and 0.01% tyloxapol. Subsequently, the iron-depleted cells were inoculated into 5 mL of the complete low-iron 7H9 medium at an initial OD600 of 0.1 with different treatments in the presence and absence of 0.5 mM ZnSO_4_, including 20 µM 2,2′-dipyridyl (DIP), 150 µM ammonium ferric citrate (the normal iron concentration in 7H9 medium), respectively. The OD600 of the cultures was determined at the indicated time points. All experiments were done in triplicate.

### Construction of recombinant mycobacterial strains

For gene overexpression, the multicopy plasmid pMV261 was used as described earlier (40). The coding sequences of *zur* and *ideR* were separately amplified and cloned into the pMV261 vector between the *EcoR* I and *Xba* I restriction sites. For their co-expression, we cloned the *ideR* promoter and coding region into the pMV261-*zur* vector between the *Xba* I and *Hind* III restriction sites. These recombinant plasmids were then transformed into *M. bovis* BCG cells to obtain the corresponding overexpression strains.

CRISPR interference (CRISPRi) strategy was carried out to construct the *ideR_KD_* strain (Table S1) as described previously (42). For simplicity and flexibility, the small-guide RNA (sgRNA) was designed to block transcription elongation by targeting the coding region of *ideR*. Briefly, the sgRNAs in this study contained a 20 bp region homologous to the non-template strand of the DNA targets before the corresponding protospacer-adjacent motif. The sgRNAs were annealed and cloned into an ATc-inducible expression vector pLJR965 between the *BsmB* I site. The recombinant plasmid was then transformed into the wild-type strain of *M. bovis* BCG to obtain the final knockdown strain. The expression of both *dcas9* and sgRNA required induction by 50 ng/mL ATc.

The *zur*-deleted strain of *M. bovis* BCG was constructed by allelic exchange using homologous recombination as previously described (40). Briefly, a pMind-derived suicide plasmid (49) carrying a hygromycin resistance gene was constructed. The 800 bp of both upstream and downstream of *zur* was successively cloned into the pMind plasmid, and then, *lacZ* gene was inserted into the plasmid as a selection marker. The final plasmid was transformed into *M. bovis* BCG and selected on 7H10 medium containing 100  µg/mL hygromycin and 40 µg/mL X-gal. To confirm the deletion of *zur*, genomic DNA from the *zur*-deleted strain and the wildtype strain was amplified by PCR. The resulting amplicons were detected by agarose gel electrophoresis and sequencing analysis. For *zur* complementation, the gene was cloned into the pMindD plasmid between the *EcoR* I and *Xba* I restriction sites. The recombinant plasmid was then transformed into the *zur*-deleted strain to obtain the *zur* complementation strain. All strains and their corresponding plasmids are listed in Table S1, and the related primers are listed in Table S2.

### Evaluation of mycobacterial growth

The growth patterns of *M. bovis* BCG strains were evaluated using modified versions of procedures as described earlier (16). Briefly, mycobacterial strains were cultured in 7H9 medium supplemented with the corresponding antibiotic until an OD600 of 1.5 was reached. Each culture was then inoculated into 50 mL of fresh 7H9 medium containing the antibiotic at an initial OD600 of 0.1 and treated with and without 0.5 mM ZnSO_4_. When required, 50 ng/mL ATc was added. The OD600 of the cultures was determined every 48 h. The zinc sensitivity of *M. bovis* BCG strains was assessed as described previously (16). Briefly, recombinant strains were grown in 7H9 medium until reaching an OD600 of 0.8 and then serially diluted. Two microliters of the dilution was spotted on 7H10 solid medium in the presence and absence of 0.5 mM ZnSO_4_ and incubated at 37°C for 2 weeks. All experiments were done in triplicate.

### Protein expression and purification

The genes *ideR* and *zur* were amplified by PCR using specific primers (Table S2). The amplified DNA fragments were digested, cloned into the pET-28a vector between the *EcoR* I and *Xba* I restriction sites, and then transformed into *E. coli* BL21 (DE3). The recombinant proteins were purified as described previously (40). After purification, the eluted His-Zur and His-IdeR were dialyzed against a low salt buffer (20 mM Tris-HCl, pH 8.0, 100 mM NaCl, 1 mM DTT, 10% glycerol) supplemented with 1 mM EDTA at room temperature and 4°C for 4 h, respectively, and then dialyzed against the low salt buffer for another 2 h. The recombinant proteins were divided into aliquots and stored at −80°C. Protein concentration was determined using the Coomassie Brilliant Blue assay.

### EMSA

The DNA-binding ability of Zur/IdeR was evaluated using a modified EMSA, as described previously (40). Briefly, reaction mixtures (20 µL) containing DNA fragments, various concentrations of proteins, and the reaction buffer (20 mM Tris-HCl at pH 8.0, 1 mM DTT, 50 mM KCl, 5 mM MgCl_2_, 0.05 mg/mL BSA, 10% glycerol) were co-incubated in the presence or absence of zinc at different concentrations. To study the effect of zinc on the DNA-binding ability of IdeR in the presence of Zur, IdeR was first incubated with the indicated concentrations of Zur at room temperature for 15 min and then treated with and without different amounts of zinc for another 15 min. Subsequently, the promoter esx-3p2 was added to the mixture and incubated for 20 min. The resulting mixtures were electrophoresed on a 6% native polyacrylamide gel containing 20 mM Tris-glycine (pH 8.0), 200 µM MnCl_2_, 10% glycerol, 1 mM DTT in the running buffer (Tris-glycine-20 mM, 200 µM MnCl_2_, and 1 mM DTT) at 150 V at 16°C. Images were recorded using an FLA-5100 Fluorescent Image Analyzer (FUJIFILM, Japan).

### GST pull-down assay

The physical interaction between Zur and IdeR was determined by GST pull-down assays with several modifications to a previous procedure (50). Briefly, GST and GST-tagged IdeR (GST-IdeR) proteins were expressed and purified from *E. coli* BL21 (DE3). After purification, the eluates were dialyzed overnight at 4°C. For the pull-down assay, approximately 100 µg of GST and GST-IdeR was separately immobilized in 50 µL of glutathione agarose and equilibrated before being incubated together at 4°C for 1 h with a gentle rocking motion. Subsequently, about 300 µg of His-Zur was added to the immobilized GST-IdeR and GST after three washes with PBST. The two fusion proteins were incubated overnight at 4°C under gentle rotation. The agaroses were washed three times with PBST, eluted with elution buffer (10 mM glutathione in PBS, pH 8.0), and then analyzed by SDS-PAGE and immunoblot assay using the antibodies indicated.

### Bacterial two-hybrid assay

The BacterioMatch II Two-Hybrid System (Stratagene) was used to detect the interaction between IdeR and Zur, as described previously (40). In brief, all recombinant strains were cultured in LB medium supplemented with 15 µg/mL tetracycline, 34 µg/mL chloramphenicol, and 50 µg/mL kanamycin until the stationary phase was reached. Two microliters of the cells was then spotted on the selective screening medium plate containing 5 mM 3-amino-1,2,4-triazole (Stratagene) and 8 µg/mL streptomycin and cultivated at 30°C for 3 days. The positive control (CK+) contained co-transformant plasmids, pBT-*LGF2* and pTRG-*Gal11p* (Stratagene), while the negative control (CK-) contained empty vectors pBT and pTRG.

### Co-immunoprecipitation assays

The interaction between Zur and IdeR in *M. bovis* BCG was analyzed by Co-IP as previously described (51) with several modifications. Briefly, IdeR with a C-terminal antigenic Flag (DDDDK) tag was cloned into the pMV261 vector between the *EcoR* I and *Xba* I restriction sites. The resulting recombinant plasmid was transformed into *M. bovis* BCG cells. The recombinant strains were then cultured in 7H9 medium until reaching an OD600 of 1.0 and were treated with and without 0.5 mM ZnSO_4_ for 24 h. After treatment, the cells were fixed with 1% formaldehyde for 20 min and the reaction was stopped with 0.125 M glycine for 5 min. Cross-linked cells were harvested, resuspended, and lysed in 5 mL of TBSTT buffer [20 mM Tris-HCl (pH 7.5), 150 mM NaCl, 0.1% Tween 20, 0.1% Triton X-100]. Co-IPs were performed by incubating and shaking 1 mL of mycobacterial cell extract with 10 µL of Zur antiserum for 1 h at 4°C. Next, 50 µL protein A Sepharose was added and incubated for another hour. The beads were then washed three times with 1 mL of the same buffer and centrifuged at 800 *g* for 1 min. Finally, the beads were resuspended in SDS-PAGE sample buffer, boiled, and analyzed by western blotting using anti-Flag antibody.

### Quantitative real-time PCR

*M. bovis* BCG strains were cultured in 7H9 medium up to an OD600 of 1.0 and then supplemented with and without 0.5 mM ZnSO_4_ for 4 or 24 h. Total RNA was extracted from the cells equivalent to 10 OD600 (e.g., 10 mL of a culture with OD600 of 1.0) using TRIzol reagent (Invitrogen), as described previously (40). The concentration and quality of total RNA were accessed by NanoDrop 2000 (Thermo Scientific, USA), treated with DNase I (Takara Biotechnology, Japan), and then reverse transcribed into cDNA using the PrimeScript RT Reagent Kit. qRT-PCR was performed with SYBR Green mix in ABI QuantStudio 3 (Applied Biosystems, USA). Relative quantification of gene expression was performed by the 2^−ΔΔ*CT*^ method and normalized to the levels of the *16 S rRNA* gene. The primers used in this study are listed in Table S2.

### Quantification of metal ion contents

The metal ion content of *M. bovis* BCG strains was determined by ICP-OES (Varian, USA) (40) or using iron-detection reagent assays (52). Briefly, the wild-type strain, the *ctpG*-deleted strain, and the *zur*-deleted strain of *M. bovis* BCG were cultured in 300  mL of 7H9 up to an OD600 of 0.8. The cultures were then treated with or without the indicated concentrations of ZnSO_4_ for 24 h. The resulting cell pellets were dried, digested, and measured following previously described procedures (40, 52).

### Siderophore purification and analysis

Siderophores containing MBT and cMBT were obtained from *M. bovis BCG* extracts sourced from high-cell-density whole cells and supernatants, respectively, as described previously (37). *M. bovis* BCG strains were cultured in 100 mL 7H9 medium until reaching an OD600 of 0.8 and then treated with and without 0.5 mM ZnSO_4_ for 24 h. After treatment, the cells and supernatants were harvested by centrifugation. To obtain MBT, the resulting cell pellets were washed once in PBS, suspended in 20 mL of 100% ethanol, and stirred overnight at room temperature. The extract was centrifuged at 8,000 rpm for 5 min, and the supernatant was mixed with one volume of chloroform. Next, 10% (wt/vol) FeCl_3_ in 100% ethanol was added dropwise to the solution until the red color saturation was observed. Subsequently, three-fourths of the volume of double distilled H_2_O was added to form a biphasic solution (chloroform and water/ethanol phases). The organic phase containing siderophores was then washed four times with one volume of H_2_O, dried with anhydrous MgSO_4_ to remove water leftovers, and finally centrifuged and evaporated.

The purified siderophores were dissolved in 100% ethanol and analyzed using an Ultimate 3000 chromatography system (Thermo Fisher Scientific, USA) with spectrophotometric detection of the eluted sample at 450 nm, as previously described (41). In brief, a reverse-phase column (50 mm × 2.1 mm, 1.7 µM) packed with ACQUITY UPLC-BEH-C18 was used. The mobile phase was initially water/formic acid (99.9:0.1 vol/vol), and the gradient used for elution was as follows: time (min)/% of methanol: 0–2 min/5%; 2–13 min/5%–100%; 13–17 min/100%; 17–17.1 min/100%–5%; 17.1–19 min/5%. The flow rate was maintained at 0.3 mL/min and performed at 30°C. The injection volume was 4 µL. The eluting species were analyzed using a Q-Exactive MS (Thermo Fisher Scientific, USA) equipped with heated electrospray ionization (HESI) source. The mass spectrometer operated in positive ionization mode, and ions scanned in the range *m*/*z* 63-945 for MBT and *m*/*z* 55-835 for cMBT. The HESI conditions were as follows: sheath gas flow rate was set at 35, the auxiliary gas flow rate at 10, spray voltage at 3.5 kV, and capillary temperature at 320°C. The settings for full scan mode were Targeted-SIM/dd-MS2 with a resolution of 70,000 FWHM (full MS) and 35,000 FWHM (data-dependent MS2). The data were processed using Thermo Xcalibur Qual Browser software (Version 2.2.0, Thermo Fisher Scientific Inc., USA).

### Prediction of sequence features and structure of Zur-IdeR

AlphaFold v2.2.0 was performed to predict the interaction between IdeR and Zur according to the previous work (44). The concrete parameters are listed as follows: model_preset = multimer; db_preset = full_dbs. A FASTA file containing the sequences of both IdeR and Zur proteins was input into the AlphaFold software. The resulting models were ranked by the AF-score and rank one was selected. The AF-score is a linear combination of the metric interface score (ipTM) and the predicted TM-score (pTM): AF-score = 0.8 ipTM + 0.2 pTM.

### NanoLC-MS analysis

Five micromolar IdeR was incubated with 10 µM Zur and the promoter esx-3p2, following the EMSA procedures mentioned above. After incubation, the mixtures were electrophoresed on a 6% native polyacrylamide gel. The proteins from the shifted band corresponding to the IdeR-DNA complex in the gel were extracted and processed according to previously published procedures (53) with modifications. The samples were digested with trypsin and vacuum concentrated to remove liquid. The resulting peptides were then centrifuged and suspended in NanoLC-MS buffer A. The supernatant was added to an injection vial and analyzed using the EASY-n LC1000 system coupled with LTQ-Orbitrap Elite mass spectrometer (Thermo Fisher, USA). The mobile phase A consisted of 0.1% formic acid and 2% acetonitrile in water, while the mobile phase B contained 0.1% formic acid and 98% acetonitrile in water. The gradient was programmed to increase or decrease linearly as follows: 2%–12% B from 0 to 5 min, 12%–20% B from 5 to 30 min, 20%–32% from 30 to 43 min, and 32%–98% from 43 to 58 min at a flow rate of 250 nL/min. The MS scan ranges were *m*/*z* 300–1,800 Da. The resolution for Orbitrap analyzer was set as 70,000 for MS and 17,500 for MS/MS. The Proteome Discoverer 2.1 software was used to search the database to enable the NanoLC-MS spectrum to match certain peptides of IdeR and Zur.

### Statistical analysis

Where indicated, statistical analyses were carried out using GraphPad Prism7.

## Data Availability

All data described are presented either within the article or in the supporting information.

## SUPPLEMENTAL MATERIAL

Fig. S1 to S15, Table S1 to S2 spectrum.01069-23-s0001.docx Supplementary Fig. 1 Zinc causes intracellular iron accumulation of the wildtype and cptG-deleted strains of M. bovis BCG. Supplementary Fig. 2 Zinc stimulates esxG/H expression in M. bovis BCG. Supplementary Fig. 3 The deletion of esxH leads to distinct orange pigmentation in M. bovis BCG. Supplementary Fig. 4 The effects of zinc on MBT and cMBT levels in the wild-type and esxH-deleted M. bovis BCG strains. Supplementary Fig. 5 IdeR inhibits the expression of iron uptake genes in M. bovis BCG. Supplementary Fig. 6 The growth difference between recombinant M. bovis BCG strains in 7H9 medium. Supplementary Fig. 7 EMSA assays for the DNA-binding ability of Zur and IdeR. Supplementary Fig. 8 EMSA assays for the effect of CmtR on in the DNA-binding ability of IdeR. Supplementary Fig. 9 EMSA assays for the effects of zinc on the DNA-binding ability of Zur and IdeR. Supplementary Fig. 10 The effect of zur deletion on the growth of M. bovis BCG in 7H9 medium. Supplementary Fig. 11 qRT-PCR assays for the regulatory effect of Zur on the expression of the IdeR regulon in M. bovis BCG. Supplementary Fig. 12 The effects of zinc on intracellular MBT and cMBT levels of the zur-deleted M. bovis BCG strain. Supplementary Fig. 13 The deletion of zur impairs M. bovis BCG growth. Supplementary Fig. 14 Phylogenetic analysis of IdeR and Zur paralogs. Supplementary Fig. 15 Co-expression of zur and ideR inhibits bacterial growth. Supplementary Table 1 Strains and plasmids used in this study. Supplementary Table 2 Primers used in this study.

ASM does not own the copyrights to Supplemental Material that may be linked to, or accessed through, an article. The authors have granted ASM a non-exclusive, world-wide license to publish the Supplemental Material files. Please contact the corresponding author directly for reuse.
