# Supplementary material for: Zinc excess impairs Mycobacterium bovis growth through triggering a Zur-IdeR-iron homeostasis signal pathway
Source: Microbiol Spectr. 2023 Sep 5;11(5):e01069-23. doi: 10.1128/spectrum.01069-23 (PMC10580935; doi:10.1128/spectrum.01069-23)
Supplement: Fig. S1 to S15, Table S1 to S2 — Supplementary Fig. 1 Zinc causes intracellular iron accumulation of the wildtype and cptG-deleted strains of M. bovis BCG. Supplementary Fig. 2 Zinc stimulates esxG/H expression in M. bovis BCG. Supplementary Fig. 3 The deletion of esxH leads to distinct orange pigmentation in M. bovis BCG. Supplementary Fig. 4 The effects of zinc on MBT and cMBT levels in the wild-type and esxH-deleted M. bovis BCG strains. Supplementary Fig. 5 IdeR inhibits the expression of iron uptake genes in M. bovis BCG. Supplementary Fig. 6 The growth difference between recombinant M. bovis BCG strains in 7H9 medium. Supplementary Fig. 7 EMSA assays for the DNA-binding ability of Zur and IdeR. Supplementary Fig. 8 EMSA assays for the effect of CmtR on in the DNA-binding ability of IdeR. Supplementary Fig. 9 EMSA assays for the effects of zinc on the DNA-binding ability of Zur and IdeR. Supplementary Fig. 10 The effect of zur deletion on the growth of M. bovis BCG in 7H9 medium. Supplementary Fig. 11 qRT-PCR assays for the regulatory effect of Zur on the expression of the IdeR regulon in M. bovis BCG. Supplementary Fig. 12 The effects of zinc on intracellular MBT and cMBT levels of the zur-deleted M. bovis BCG strain. Supplementary Fig. 13 The deletion of zur impairs M. bovis BCG growth. Supplementary Fig. 14 Phylogenetic analysis of IdeR and Zur paralogs. Supplementary Fig. 15 Co-expression of zur and ideR inhibits bacterial growth. Supplementary Table 1 Strains and plasmids used in this study. Supplementary Table 2 Primers used in this study. [file spectrum.01069-23-s0001.docx]

**Zinc excess impairs *Mycobacterium bovis* growth through triggering a Zur-IdeR-Iron homeostasis signal pathway**

**Xiaohui Li,^a^ Liu Chen,^b^ Yuankun Wang,^a^ Xiao Guo,^a^ and** **Zheng-Guo He****^a^**^#^

^a^State Key Laboratory for Conservation and Utilization of Subtropical Agro-bioresources, Guangxi Research Center for Microbial and Enzyme Engineering Technology, College of Life Science and Technology, Guangxi University, Nanning 530004, China.

^b^College of Life Science and Technology, Huazhong Agricultural University, Wuhan 430070, China.

^#^Address correspondence to Zheng-Guo He, hezhengguo2019@163.com

Tel: +86-771-3225146, Fax: +86-771-3225146

**Running title:** A novel antimycobacterial pathway of zinc toxicity

**SUPPLEMENTAL MATERIAL**

**Supplementary Fig. 1** Zinc causes intracellular iron accumulation of the wildtype and *cptG*-deleted strains of *M. bovis* BCG.

**Supplementary Fig. 2** Zinc stimulates *esxG/H* expression in *M. bovis* BCG.

**Supplementary Fig. 3** The deletion of *esxH* leads to distinct orange pigmentation in *M. bovis* BCG.

**Supplementary Fig. 4** The effects of zinc on MBT and cMBT levels in the wild-type and *esxH*-deleted *M. bovis* BCG strains.

**Supplementary Fig. 5** IdeR inhibits the expression of iron uptake genes in *M. bovis* BCG.

**Supplementary Fig. 6** The growth difference between recombinant *M. bovis* BCG strains in 7H9 medium.

**Supplementary Fig. 7** EMSA assays for the DNA-binding ability of Zur and IdeR.

**Supplementary Fig. 8** EMSA assays for the effect of CmtR on in the DNA-binding ability of IdeR.

**Supplementary Fig. 9** EMSA assays for the effects of zinc on the DNA-binding ability of Zur and IdeR.

**Supplementary Fig. 10** The effect of *zur* deletion on the growth of *M. bovis* BCG in 7H9 medium.

**Supplementary Fig. 11** qRT-PCR assays for the regulatory effect of Zur on the expression of the IdeR regulon in *M. bovis* BCG.

**Supplementary Fig. 12** The effects of zinc on intracellular MBT and cMBT levels of the *zur*-deleted *M. bovis* BCG strain.

**Supplementary Fig. 13** The deletion of *zur* impairs *M. bovis* BCG growth.

**Supplementary Fig. 14** Phylogenetic analysis of IdeR and Zur paralogs.

**Supplementary Fig. 15** Co-expression of *zur* and *ideR* inhibits bacterial growth.

**Supplementary Table 1** Strains and plasmids used in this study.

**Supplementary Table 2** Primers used in this study.


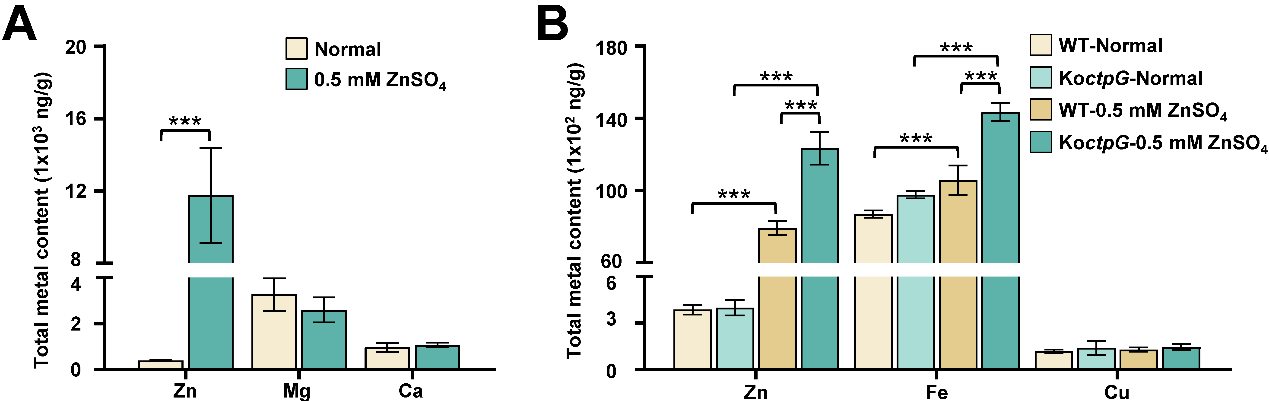


**Supplementary Fig. 1** **Zinc causes intracellular iron accumulation of the wildtype and *cptG*-deleted strains of *M. bovis* BCG.** The wildtype strain (**A** and **B**) and the *ctpG*-deleted strain (**B**) were cultured in 7H9 medium up to an OD600 of 0.8 and then treated with or without 0.5 mM ZnSO_4_ for 24 h. Subsequently, the intracellular metal content in bacterial pellets was determined using ICP-OES**.** *Error bars*, S.D. Statistical significance was calculated with Student’s *t*-test; ***, *P* < 0.001.


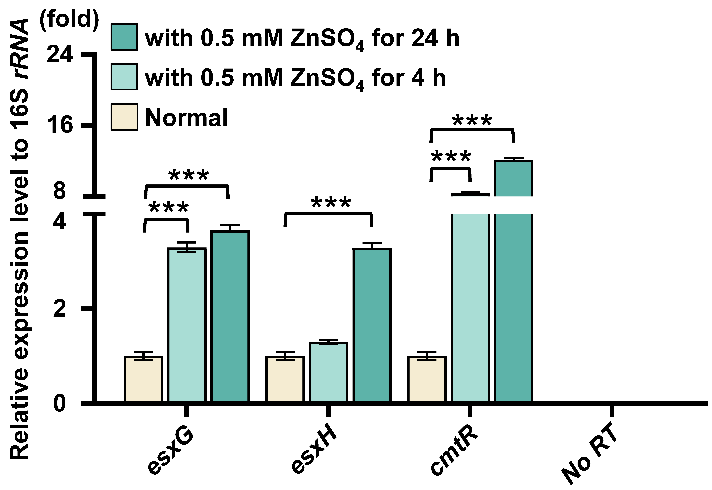


**Supplementary Fig. 2** **Zinc stimulates *esxG/H* expression in *M. bovis* BCG.** qRT-PCR analysis of *esxG/H* expression in *M. bovis* BCG strain upon exposure to 0.5 mM zinc for 4 h or 24 h. The untreated strain and the reported zinc-induced gene (*cmtR*) (1) were separately used as controls. *Error bars*, S.D. Statistical significance was calculated with Student’s *t*-test; ***, *P* < 0.001.


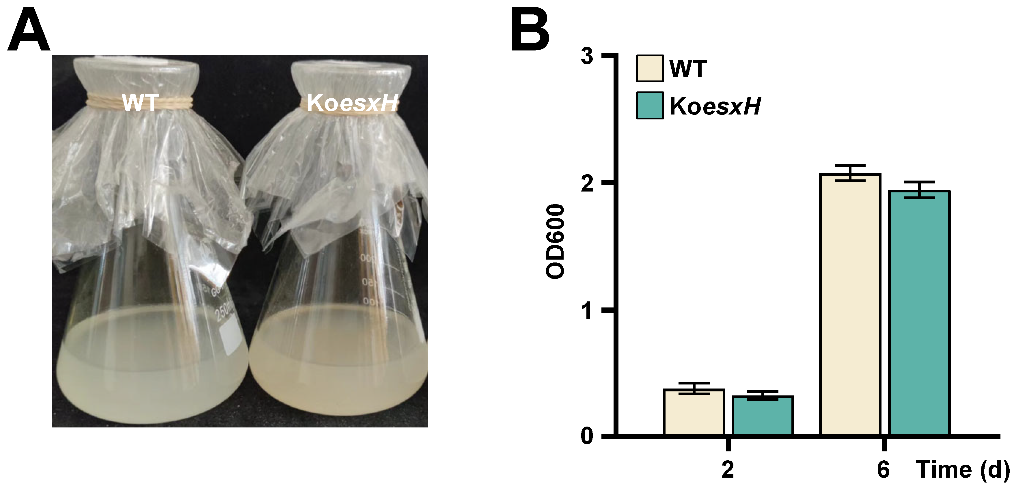


**Supplementary Fig. 3 The deletion of *esxH* leads to distinct orange pigmentation in *M. bovis* BCG.** (**A**) The coloration difference between the wild-type strain and the *esxH*-deleted strain (KO*esxH*). *M. bovis* BCG strains were cultured in 7H9 medium to stationary phase, and then the culture coloration was photographed. (**B**) The growth difference between the wild-type and *esxH*-deleted strains in 7H9 medium. *Error bars*, S.D. Statistical significance was calculated with Student’s *t*-test.


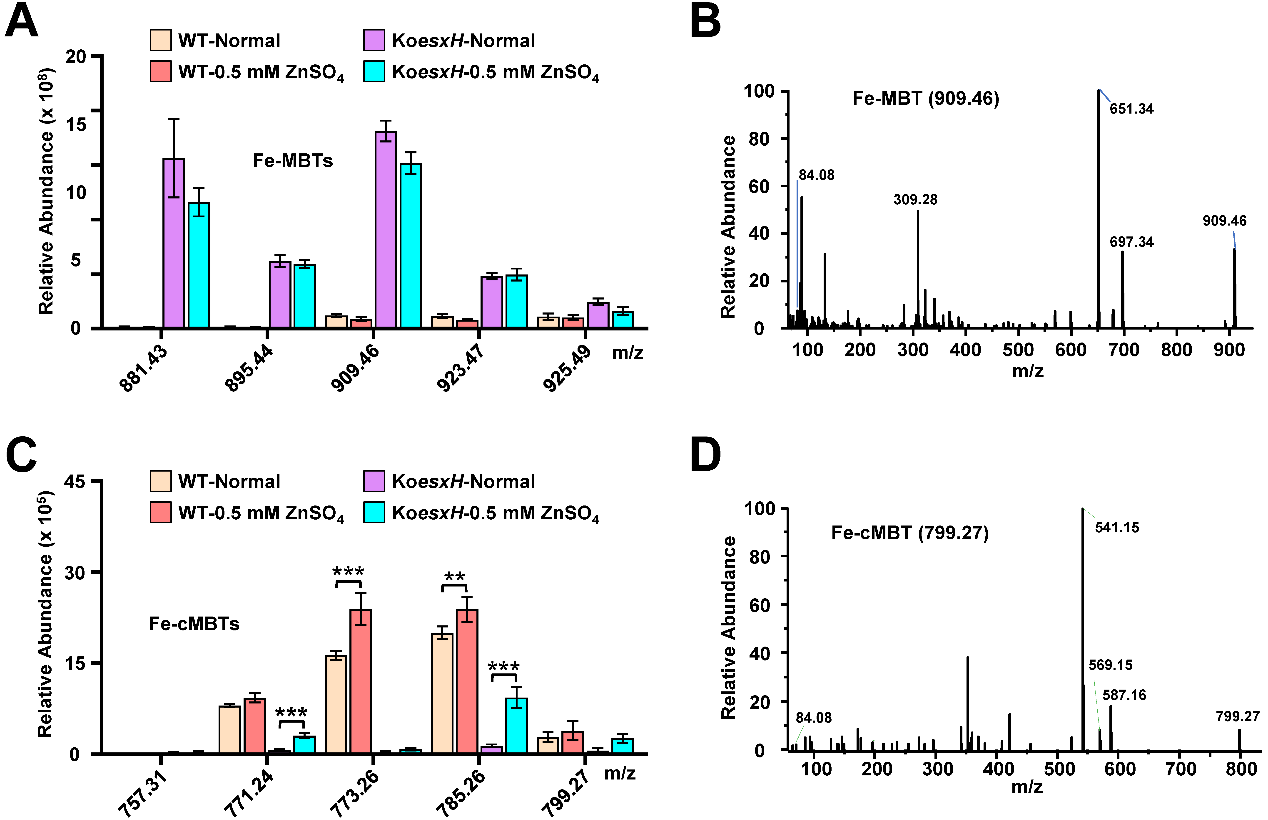


**Supplementary Fig. 4 The effects of zinc on MBT and cMBT levels in the wild-type and *esxH*-deleted *M. bovis* BCG strains.** (**A** and **C**) MBT (**A**) and cMBT (**C**) abundance in whole-cell extracts of the wild-type strain (WT) and the *esxH*-deleted strain treated with and without 0.5 mM zinc stress. (**B** and **D**) Q-Exactive mass spectrum of the 909 m/z ion for Fe-MBT (**B**) and the 799 m/z ion for Fe-cMBT (**D**) from the extracts of the *esxH*-deleted strain, respectively. *Error bars*, S.D. Statistical significance was calculated with Student’s *t*-test; **, *P* < 0.01; ***, *P* < 0.001.


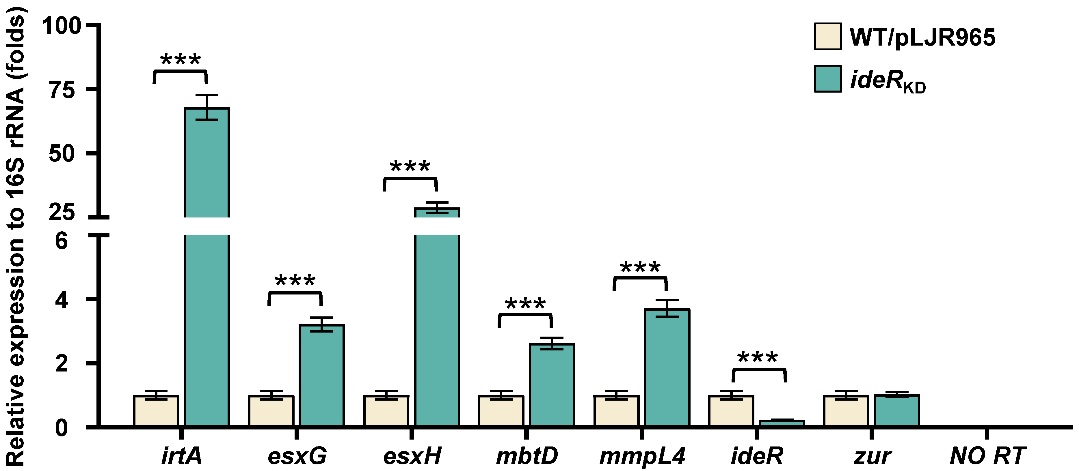


**Supplementary Fig. 5 IdeR inhibits the expression of iron uptake genes in *M. bovis* BCG.** qRT-PCR analysis of the effect of *ideR* silencing on the expression of iron uptake genes in *M. bovis* BCG. Recombinant strains mentioned in Fig. 3A were cultured in 7H9 medium up to an OD600 of 1.0 and then treated with 50 ng/mL ATc for 24 h. subsequently, the cells were harvested for qRT-PCR assays. *Error bars*, S.D. Statistical significance was calculated with Student’s *t*-test; ***, *P* < 0.001.


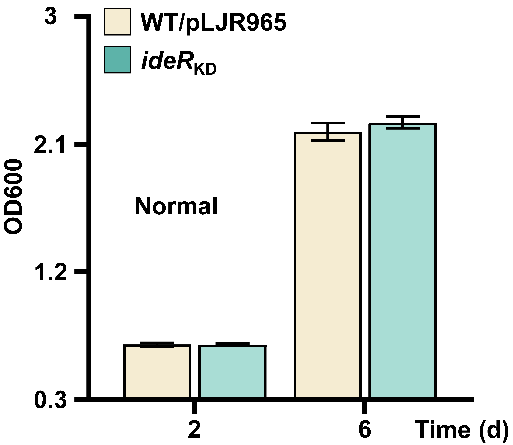


**Supplementary Fig. 6 The growth difference between recombinant *M. bovis* BCG strains in 7H9 medium.** Recombinant strains mentioned in Fig. 3A were cultured in 7H9 medium at an initial OD600 of 0.1, and their growth difference was determined at the indicated time point.


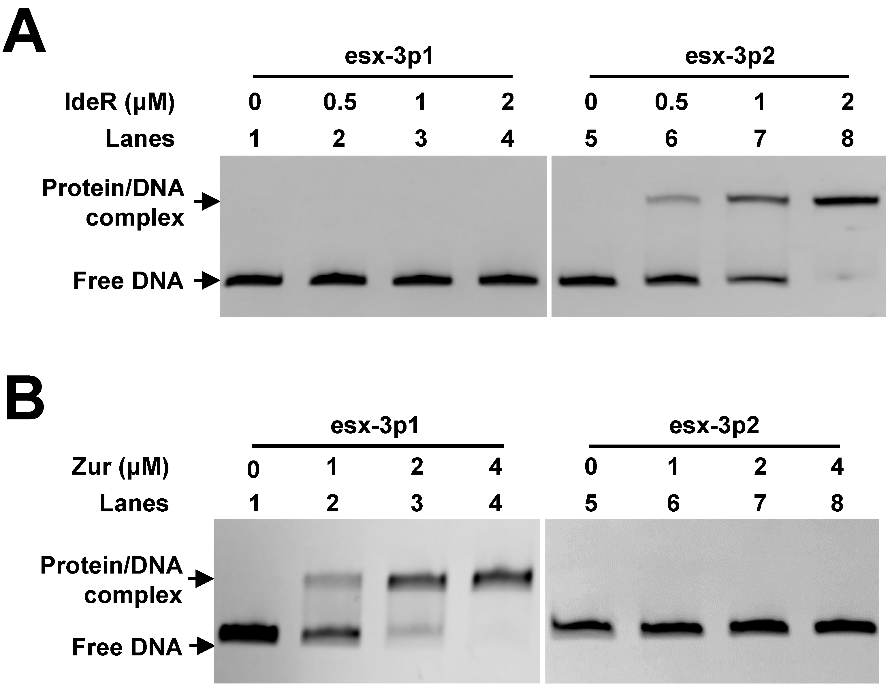


**Supplementary Fig. 7 EMSA assays for the DNA-binding ability of Zur and IdeR. (A** and **B**) The increasing concentrations of IdeR (**A**) and Zur (**B**) were separately incubated with the *esx3* operon promoters, esx3-p1 (lanes 1-4) and esx3-p2 (lanes 5-8).


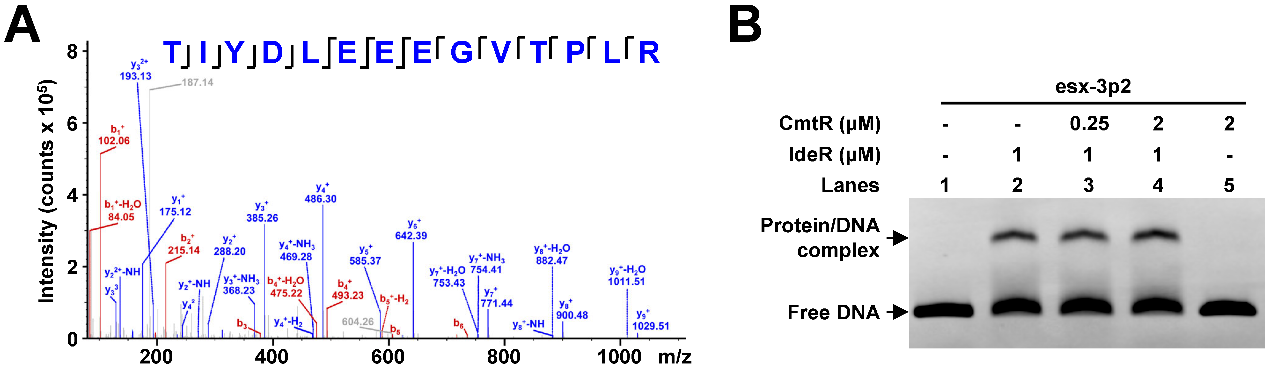


**Supplementary Fig. 8** **EMSA assays for the effect of CmtR on in the DNA-binding ability of IdeR.** (**A**) NanoLC-MS identified the peptide of IdeR in the shifted band corresponding to the IdeR-Zur-DNA complex, whose amino acid sequence was TIYDLEEEGVTPLR. (**B**) IdeR were incubated with the promoter esx-3p2 in the presence and absence of different concentrations of CmtR (0.25-2 μM).


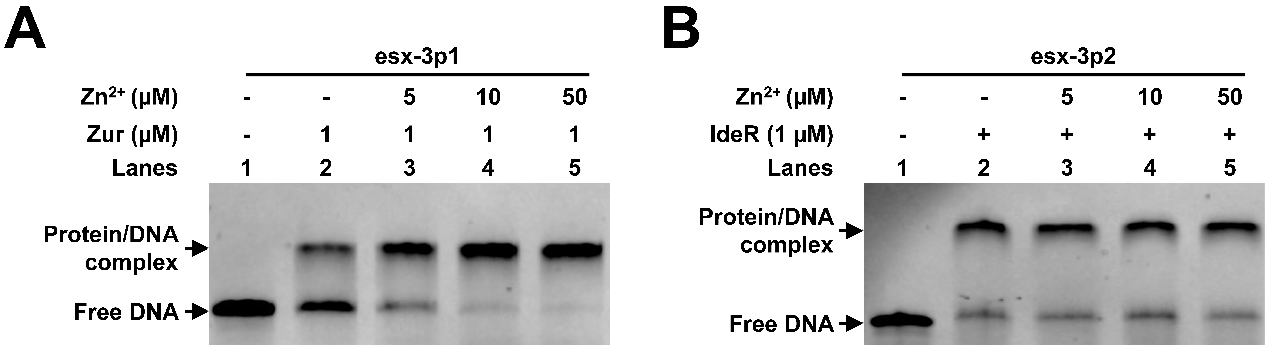


**Supplementary Fig. 9** **EMSA assays for the effects of zinc on the DNA-binding ability of Zur and IdeR.** Zur (**A**) and IdeR (**B**) were separately incubated with the corresponding promoter, including esx3-p1 and esx3-p2, in the presence and absence of zinc at different concentrations.


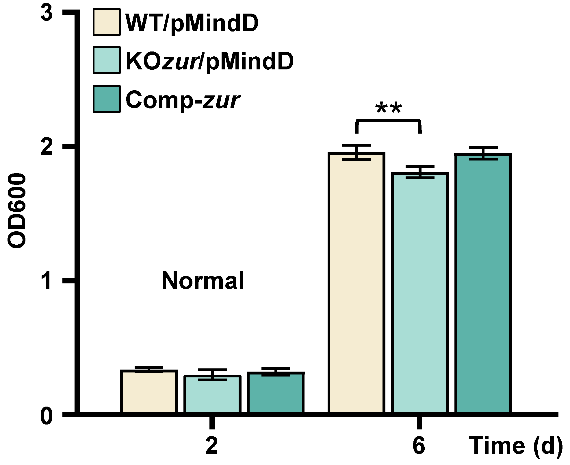


**Supplementary Fig. 10 The effect of *zur* deletion on the growth of *M. bovis* BCG in 7H9 medium.** Recombinant strains mentioned in Fig. 5A were cultured in 7H9 medium at an initial OD600 of 0.1, and their growth difference was determined. *Error bars*, S.D. Statistical significance was calculated with Student’s *t*-test; **, *P* < 0.01.


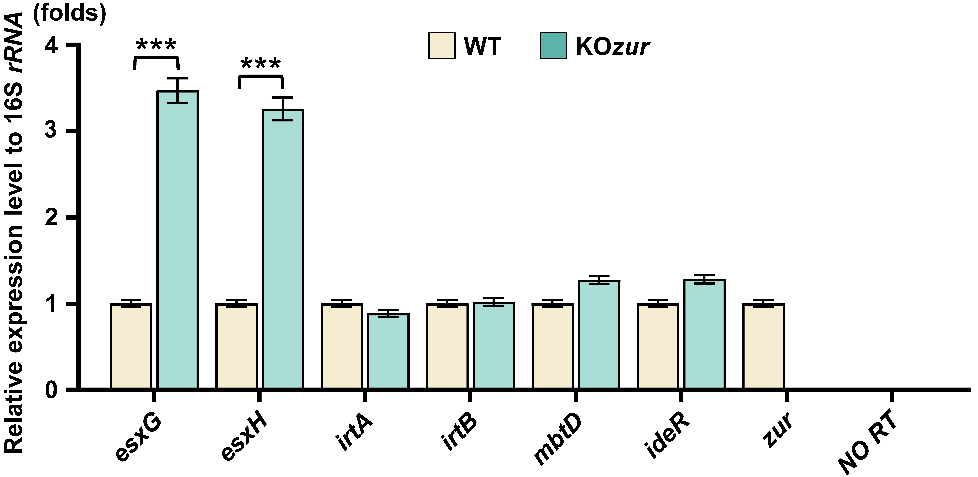


**Supplementary Fig. 11 qRT-PCR assays for the regulatory effect of Zur on the expression of the IdeR regulon in *M. bovis* BCG**. The wildtype strain and the zur-deleted strain were cultured in 7H9 medium until reaching an OD600 of 1.0 and then harvested for qRT-PCR assays. WT represents the BCG/WT strain; KO*zur* represents the BCG/*zur*::*hyg* strain. *Error bars*, S.D. Statistical significance was calculated with Student’s *t*-test; ***, *P* < 0.001.


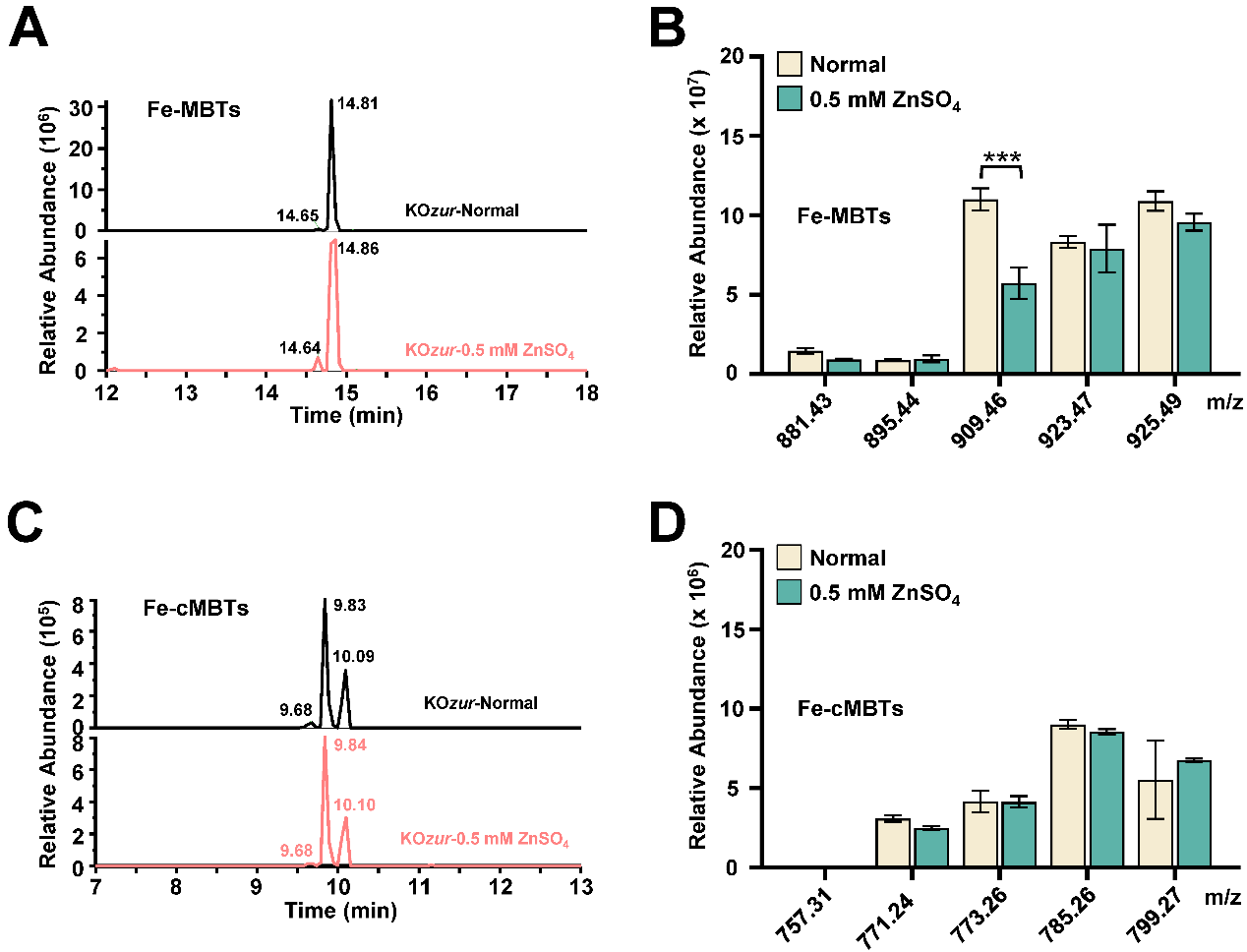


**Supplementary Fig. 12 The effects of zinc on intracellular MBT and cMBT levels of the *zur*-deleted *M. bovis* BCG strain.** (**A**-**D**) MBT (**A**-**B**) and cMBT (**C-D**) abundance in whole-cell extracts of the *zur*-deleted strain treated with and without 0.5 mM zinc stress. *Error bars*, S.D. Statistical significance was calculated with Student’s *t*-test; ***, *P* < 0.001.


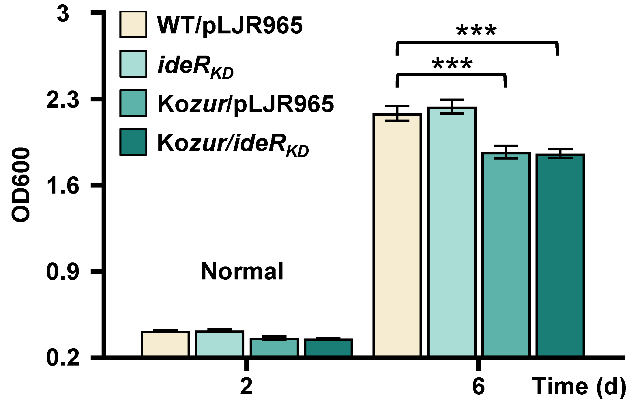


**Supplementary Fig. 13 The deletion of *zur* impairs *M. bovis* BCG growth.** Recombinant strains mentioned in Fig. 6E were cultured in 7H9 medium at an initial OD600 of 0.1, and their growth difference was determined. *Error bars*, S.D. Statistical significance was calculated with Student’s *t*-test; ***, *P* < 0.001.


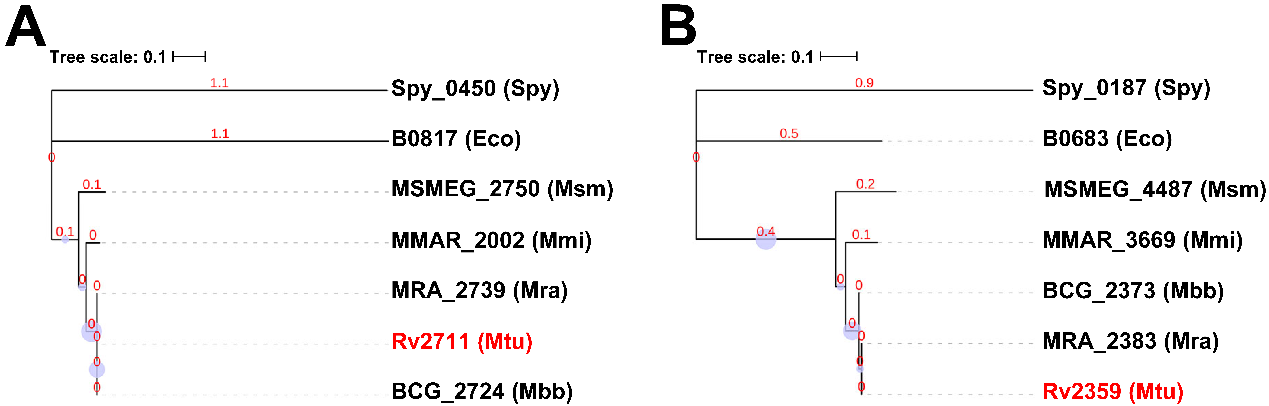


**Supplementary Fig. 14 Phylogenetic analysis of IdeR and Zur paralogs.**

Phylogenetic analysis of IdeR (**A**) and Zur (**B**) paralogs. Phylogeny was constructed using the Neighbor-Joining method of the MEGA X software (bootstrap: 10000 replicates, bootstrap values indicated by circle sizes). Protein sequences are obtained from the GeneBank database: Rv2359 (Zur) and Rv2711 (IdeR) from *M. tuberculosis* H37Rv (Mtu); BCG_2373 and BCG_2724 from *M. bovis* BCG; MRA_2739 and MRA_2383 from *M. tuberculosis* H37Ra (Mra); MMAR_2002 and MMAR_3669 from *M. marinum* (Mmi); MSMEG_2750 and MSMEG_4487 from *M. smegmatis* (Msm); B0683 and B0817 from *E. coli* K-12 (Eco); Spy_0187 and Spy_0450 from *Streptococcus pyogenes* M1 (Spy).


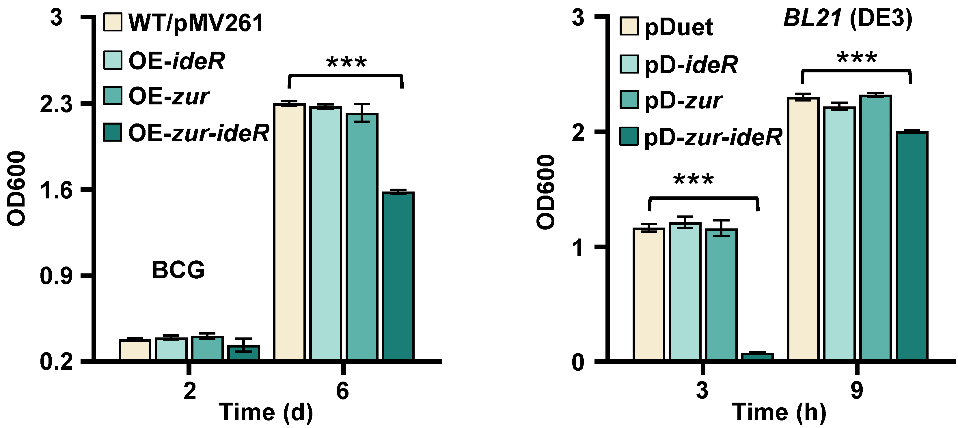


**Supplementary Fig. 15 Co-expression of *zur* and *ideR* inhibits bacterial growth.** Recombinant strains of *M. bovis* BCG or *E. coli* (DE3) were cultured in the corresponding medium at an initial OD600 of 0.1 and 0.01, respectively, and their growth difference was determined. OE-*zur* represents the BCG/pMV261-*zur* strain; OE-*zur-ideR* represents the BCG/pMV261-*zur-ideR* strain; pDuet represents the BL21 (DE3)/pRSFDuet1 strain; pD-*ideR* represents the BL21 (DE3)/pRSFDuet1-*ideR* strain; pD-*zur* represents the BL21 (DE3)/pRSFDuet1-z*ur* strain; pD-*zur-ideR* represents the BL21 (DE3)/pRSFDuet1-z*ur-ideR* strain. *Error bars*, S.D. Statistical significance was calculated with Student’s *t*-test; ***, *P* < 0.001.

**Supplementary Table 1 Strains and plasmids used in this study.**

| **Plasmid or Strain** | **Relevant genotype or feature** | **Source or reference** |
| --- | --- | --- |
| Plasmid |  |  |
| pET28a | Kan^r^, *lacZ* operon, T7 promotor, His-Tag | Novagen |
| pET28a-*ideR* | *ideR* inserted in *EcoRI-XbaI* of pMV261 | This study |
| pET28a-*cmtR* | *cmtR* inserted *in EcoRI-XbaI* of pMV261 | Previous study^1^ |
| pET28a-Sumo | Kan^r^, *lacZ* operon, T7 promotor, His-Tag (N), N-Sumo | Novagen |
| pET28a-Sumo-*zur* | *zur* inserted in *BamHI-HindIII* of pET28a-Sumo | Previous study^1^ |
| pGEX-4T-1 | Amp^r^, *lacZ* operon, Tac promotor, Gst-Tag | Amersham |
| pGEX-4T-*ideR* | *ideR* inserted in *BamHI-NotI* of pGEX-4T-1 | This study |
| pRSFDuet1 | Kan^r^, *lacZ* operon, T7 promotor, His-Tag (N) | Novagen |
| pRSFDuet1-*zur* | *zur* inserted in *BamHI-HindIII* of pRSFDuet1 | This study |
| pRSFDuet1-*ideR* | *ideR* inserted in *NdeI-XhoI* of pRSFDuet1 | This study |
| pRSFDuet1-*zur*-*ideR* | *zur* and *ideR* separately inserted in *BamHI-HindIII* and *NdeI-XhoI* of pRSFDuet1 | This study |
| pLJR965 | Kan^r^, *Sth1* sgRNA scaffold and *dCas9*, TetR, L5 attP and Int | Addgene |
| pLJR965-*ideR* | *ideR* sgRNA inserted in *BsmBI-BsmBI* of pLJR965 | This study |
| PMindD | Kan^r^, *tetR*, *pAL5000* replicon | This study |
| pMindD-*zur* | *zur* inserted in *EcoRI-XbaI* of pMindD | This study |
| pMV261 | Kan^r^, *pAL5000* replicon | This study |
| pMV261-*ideR* | *ideR* inserted in *EcoRI-XbaI* of pMV261 | This study |
| pMV261-*zur* | *zur* inserted in *EcoRI-XbaI* of pMV261 | This study |
| pMV261-*zur*-*ideR* | *zur* and ideRp*_ideR* separately inserted in *EcoRI-XbaI* and *XbaI-HindIII* of pMV261 | This study |
| Strain |  |  |
| BL21(DE3) | Host for protein expression | TaKaRa |
| *M.bovis* BCG |  |  |
| WT | *M. bovis* BCG wild-type | ATCC |
| KO*esxH* | BCG with *esxH* replaced by *hyg* | Previous study^1^ |
| KO*zur* | BCG with *zur* replaced by *hyg* | This study |
| WT/pLJR965 | BCG with pLJR965 | This study |
| Ko*zur*/pLJR965 | KO*zur* with pLJR965 | This study |
| *ideR*_KD_ | BCG with pLJR965-*ideR* | This study |
| KO*zur*/pLJR965 | KO*zur* with pLJR965 | This study |
| KO*zur*/*ideR*_KD_ | KO*zur* with pLJR965-*ideR* | This study |
| WT/pMindD | BCG with pMindD | This study |
| KO*zur*/pMindD | KO*zur* with pMindD | This study |
| Comp-*zur* | KO*zur* with pMindD-*zur* | This study |
| WT/pMV261 | BCG with pMV261 | This study |
| KO*zur*/pMV261 | KO*zur* with pMV261 | This study |
| KO*zur*/pMV261*-ideR* | KO*zur* with pMV261-*ideR* | This study |
| OE-*ideR* | BCG with pMV261-*ideR* | This study |

| ***Continued*** |  |  |
| --- | --- | --- |
| **Plasmid or Strain** | **Relevant genotype or feature** | **Source or reference** |
| OE-*zur* | BCG with pMV261-*zur* | This study |
| OE-*zur*-*ideR* | BCG with pMV261-*zur*-*ideR* | This study |
| pDuet | BL21(DE3) with pRSFDuet1 | This study |
| pDuet-*zur* | BL21(DE3) with pRSFDuet1-*zur* | This study |
| pDuet-*ideR* | BL21(DE3) with pRSFDuet1-*ideR* | This study |
| pDuet-*zur*-*ideR* | BL21(DE3) with pRSFDuet1-*zur-ideR* | This study |

**Supplementary Table 2 Primers used in this study.**

| **Usage** | **Construct Primer name** | **Sequence (from 5' to 3')** |
| --- | --- | --- |
| Cloning and expression | *ideR*-F | CCTGGAATTCCCATGAACGAGTTGGTTGATAC |
|  | *ideR*-R | TCGTGCTCTAGATCAGACTTTCTCGACCTTGA |
|  | *ideR*-F2 | GGGGGGATCCATGAACGAGTTGGTTGATAC |
|  | *ideR*-R2 | TTTAGCGGCCGCTTAGACTTTCTCGACCTTGAC |
|  | *ideR*-F3 | GGGGCATATGAACGAGTTGGTTGATAC |
|  | *ideR*-R3 | TTGACTCGAGTTAGACTTTCTCGACCTTGAC |
|  | *ideR*-F4 | CCCCCCATTCTAGAATGAACGAGTTGGTTGATAC |
|  | *ideR*-R4 | CCCGAAAGCTTTCAGACTTTCTCGACCTTGA |
|  | ideRp-F | TTTTTCTCTCTAGAGTGAGCACCAGGTGATCCG |
|  | *ideR-flag*-F | AATTGAATTCAAATGAACGAGTTGGTTGATAC |
|  | *ideR-flag*-R | AAAATCTAGATTACTTATCGTCGTCATCCTTGTAATCGACTTTCTCGACCTTGACC |
|  | *zur*-F | ATATGGATCCATGAGTGCAGCCGGTGTC |
|  | *zur*-R | CTAGAAGCTTTTAGCTCCGGCAGTCTGAG |
|  | *zur-*F2 | ATCTGAATTCAGATGAGTGCAGCCGGTGTCCG |
|  | *zur*-R2 | TTAGTCTAGACTAGCTCCGGCAGTCTGAG |
| CRISPRi | *ideR*-sgRNA-F | GGGACGGCAACCCGATGACATCGA |
|  | *ideR*-sgRNA-R | AAACTCGATGTCATCGGGTTGCCG |
| EMSA | esx-3p1-F | ACTGGCTAAATCCGTTGCCG |
|  | esx-3p1-R | AGCCGTAAACACCACAGAGG |
|  | esx-3p2-F | CTACACTCTGCATATCGGGC |
|  | esx-3p2-R | ACGTCAGCGTCTACCTTGC |
| qRT-PCR | *16S rRNA*-RT-F | GATACGGGCAGACTAGAGTA |
|  | *16S rRNA*-RT-R | GGGTATCTAATCCTGTTCGC |
|  | *esxG*-RT-F | CGGCTCAGGCGTTTCAC |
|  | *esxG*-RT-R | CCGCCGCCACAAACC |
|  | *esxH*-RT-F | CAGGCCGCGTTGCA |
|  | *esxH*-RT-R | CTGCCACGCCTGATACGT |
|  | *cmtR*-RT-F | TGCTGGATGGCGTTTGCT |
|  | *cmtR*-RT-R | GGCCCTCATAGGTTGCGACTA |
|  | *irtA*-RT-F | GTTGTATTGCATGACCCGGC |
|  | *irtA*-RT-R | CGAGTCGCCGATTAGCAGAT |
|  | *mbtD*-RT-F | TATCACCGATCCCACCCAGT |
|  | *mbtD*-RT-R | ATCACCGAGGAACACAGCAG |
|  | *mmpL4*-RT-F | CAGCTACAACGACCGTGACT |
|  | *mmpL4*-RT-R | GTGGTCCATCGTCGTTCCTT |
|  | *ideR*-RT-F | GAGTAACCGTCGAAACCAC |
|  | *ideR*-RT-R | CAGACTTTCTCGACCTTGAC |
|  | *zur*-RT-F | CTTTCGTTCGGCCCAGGAA |
|  | *zur*-RT-R | GAAGATCTCGATGGTGTGGCT |

**REFERENCES**

1. Li XH, Chen L, Liao JJ, Hui JC, Li WH, He ZG. 2020. A novel stress-inducible CmtR-ESX3-Zn^2+^ regulatory pathway essential for survival of *Mycobacterium bovis* under oxidative stress. *J Biol Chem* 295:17083-17099.
